# Supplementary material for: Three SARS-CoV-2 spike protein variants delivered intranasally by measles and mumps vaccines are broadly protective
Source: Nat Commun. 2024 Jul 3;15:5589. doi: 10.1038/s41467-024-49443-2 (PMC11222507; doi:10.1038/s41467-024-49443-2)
Supplement: Supplementary file 1 — Supplementary Information [file 41467_2024_49443_MOESM1_ESM.pdf]

Three SARS-CoV-2 spike protein variants delivered intranasally by measles and mumps vaccines are broadly protective

Yuexiu Zhang[1‡], Michelle Chamblee[1‡], Jiayu Xu[1‡], Panke Qu[1], Mohamed M. Shamseldin[2,3,4], Sung J. Yoo[1], Jack Misny[5], Ilada Thongpan[5], Mahesh KC[5], Jesse M. Hall[2], Yash A. Gupta[2], John P. Evans[1], Mijia Lu[1], Chengjin Ye[6], Cheng Chih Hsu[1], Xueya Liang[1], Luis Martinez-Sobrido[6], Jacob S. Yount[2,7], Prosper N. Boyaka[1,7], Shan-Lu Liu[1,2,7,8], Purnima Dubey[2,7], Mark E. Peeples[5, 7, 9], Jianrong Li[1,7]*

[1]Department of Veterinary Biosciences, The Ohio State University, Columbus, OH, USA
[2]Department of Microbial Infection and Immunity, College of Medicine, The Ohio State University, Columbus, OH, USA
[3]Department of Microbiology, The Ohio State University, Columbus, OH, USA
[4]Department of Microbiology and Immunology, Faculty of Pharmacy, Helwan University, Ain Helwan, Helwan, Egypt
[5]Center for Vaccines and Immunity, Abigail Wexner Research Institute at Nationwide Children's Hospital, Columbus, OH, USA
[6]Texas Biomedical Research Institute, San Antonio, TX, USA
[7]Infectious Disease Institute, The Ohio State University, Columbus, OH, USA
[8]Center for Retrovirus Research, The Ohio State University, Columbus, OH, USA
[9]Department of Pediatrics, College of Medicine, The Ohio State University, Columbus, OH, USA

‡ These authors contributed equally

*Corresponding author: Jianrong Li; email: li.926@osu.edu

Supplementary Information

Supplementary Figures and Legends 1-21

Supplementary Table 1-3

Supplementary Discussion

Supplementary References

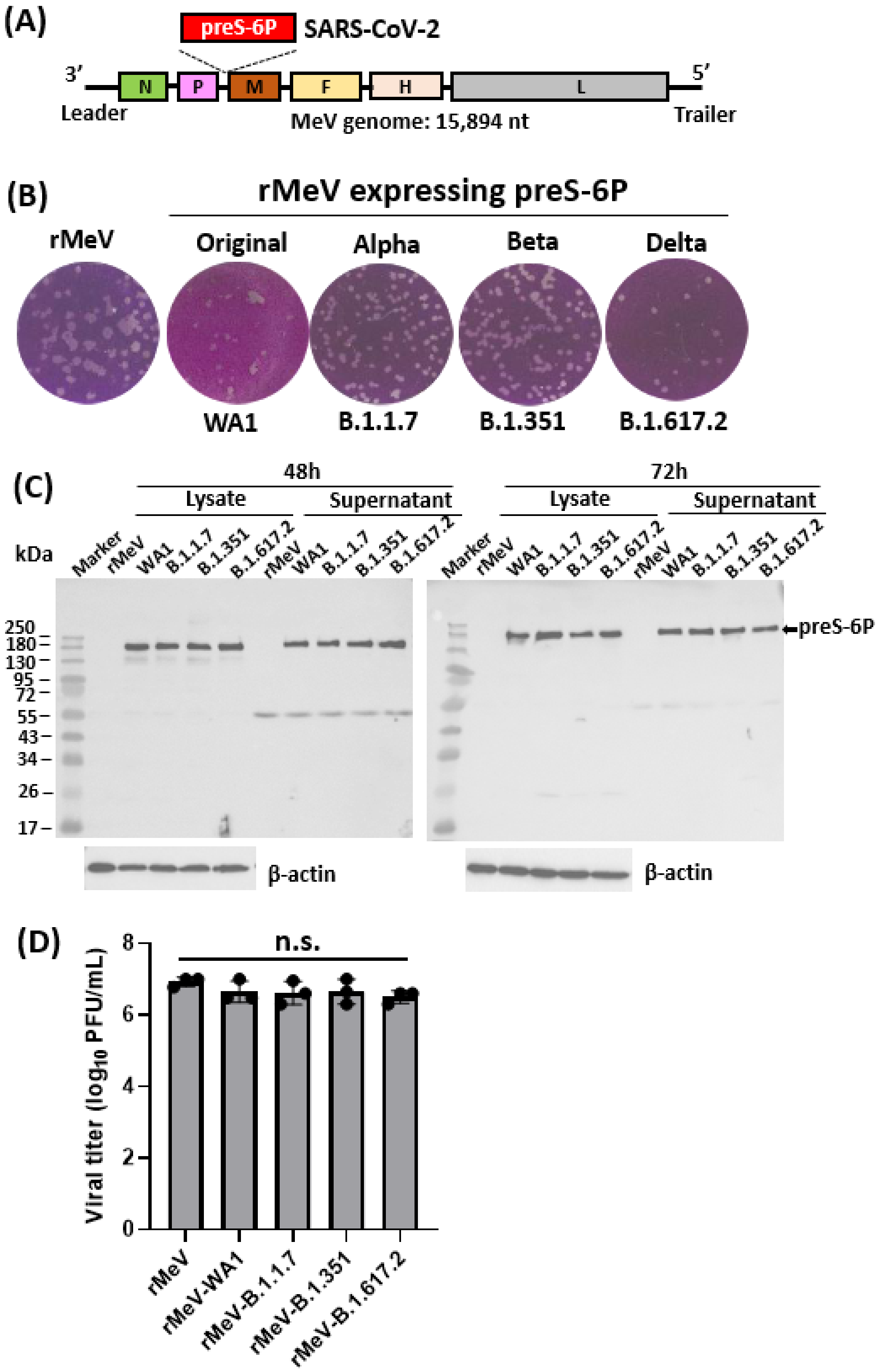

(A)
preS-6P
SARS-CoV-2
3′
N
P
M
F
H
L
5′
Leader
MeV genome: 15,894 nt
Trailer
(B)
rMeV expressing preS-6P
rMeV
Original
Alpha
Beta
Delta
WA1
B.1.1.7
B.1.351
B.1.617.2
(C)
48h
72h
Lysate
Supernatant
kDa
Marker
rMeV
WA1
B.1.1.7
B.1.351
B.1.617.2
250
180
130
95
72
55
43
34
26
17
preS-6P
β-actin
(D)
n.s.
Viral titer (log10 PFU/mL)
8
6
4
2
0
rMeV
rMeV-WA1
rMeV-B.1.1.7
rMeV-B.1.351
rMeV-B.1.617.2

Fig.S1. Characterization of rMeV expressing preS-6P of SARS-CoV-2 WA1 and variants of concern. (A) Diagram of insertion of preS-6P into the MeV genome. The *preS-6P* of SARS-CoV-2 WA1 and VoCs is inserted at the P-M gene junction in the MeV Edmonston vaccine strain. A diagram of the negative-sense RNA genome of MeV encoding 3' leader sequence, N, P, M, F, H, L, and 5' trailer sequence is shown. **(B) Plaque morphology of recombinant MeV**. The plaque assay was conducted in Vero CCL81 cells and developed after 4 days of incubation at 37ºC. **(C) Western blot analysis of preS-6P expression by rMeV**. Vero CCL81 cells in 12-well-plates were infected by each recombinant virus at an MOI of 0.1. At 48 and 72h, 10 µl of cell culture supernatant (from a total of 1 ml) and 10 µl of cell lysate (from a total of 200 µl) were analyzed by Western blot using an antibody-specific for SARS-CoV-2 S. **(D) Viral titer of each recombinant virus**. Confluent Vero CCL81 in 12-well-plates were infected with each rMeV at an MOI of 0.1. When maximum cytopathic effects (CPE) was observed, cell culture supernatant and lysate were collected for virus titration by plaque assay.

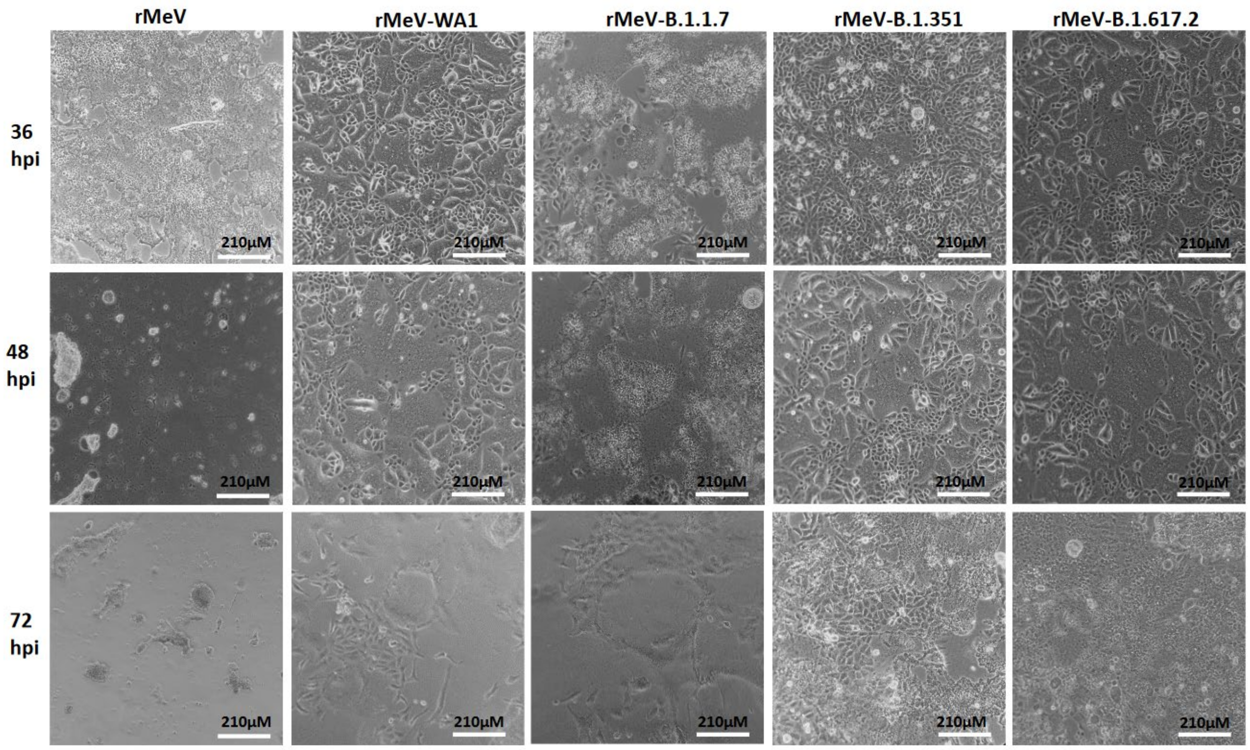

Fig.S2. Dynamics of cytopathic effects in Vero CCL81 infected by each recombinant MeV. Confluent Vero CCL81 in 12-well-plates were infected with each rMeV at an MOI of 0.1. At 36, 48, and 72 h post-infection, representative images of cytopathic effects (CPE) in cells infected by each virus are shown.

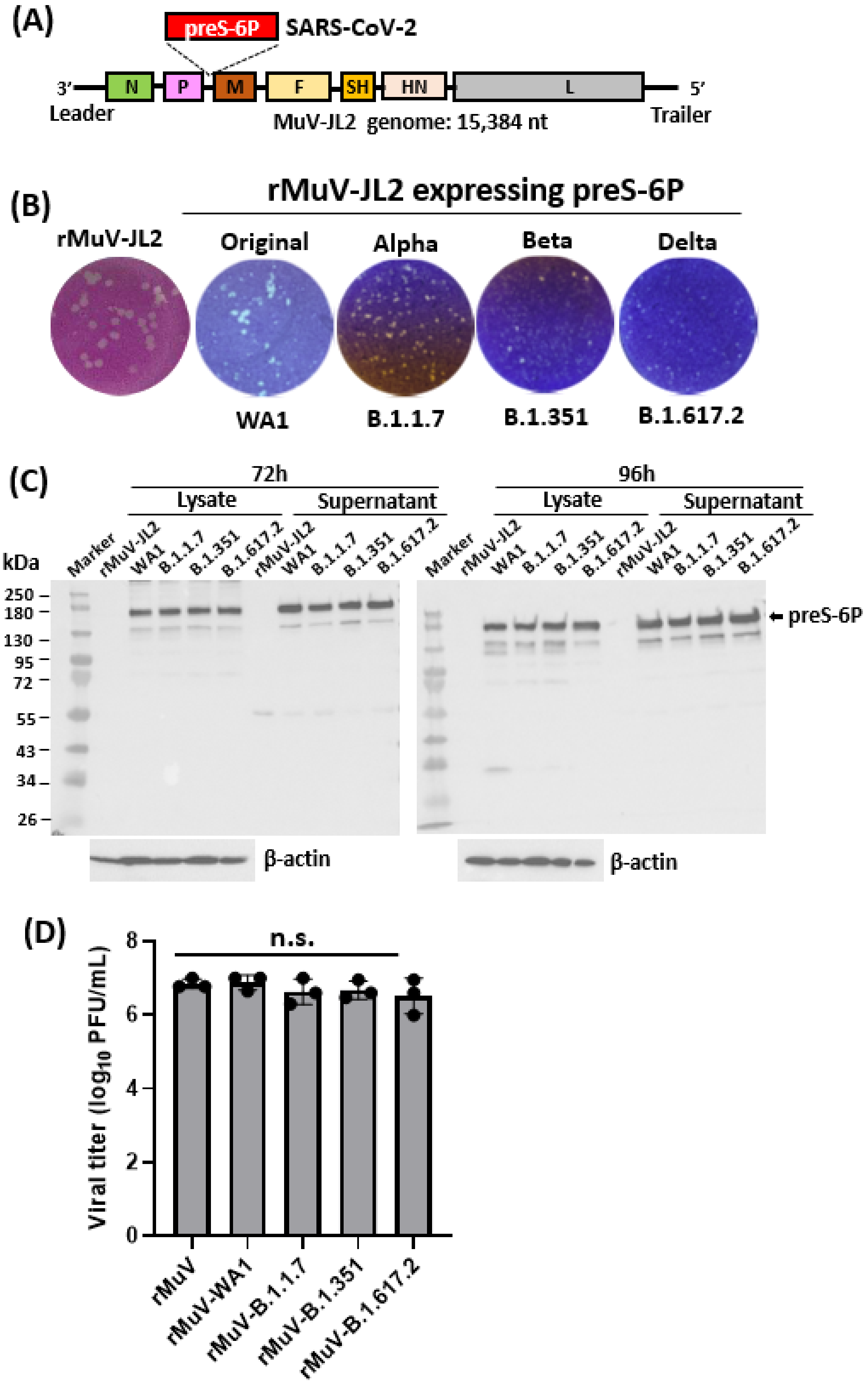
(A)
preS-6P
SARS-CoV-2
3'
N
P
M
F
SH
HN
L
5'
Leader
MuV-JL2 genome: 15,384 nt
Trailer
(B)
rMuV-JL2 expressing preS-6P
rMuV-JL2
Original
Alpha
Beta
Delta
WA1
B.1.1.7
B.1.351
B.1.617.2
(C)
72h
96h
Lysate
Supernatant
Marker
rMuV-JL2
WA1
B.1.1.7
B.1.351
B.1.617.2
kDa
250
180
130
95
72
55
43
34
26
preS-6P
β-actin
(D)
n.s.
Viral titer (log10 PFU/mL)
8
6
4
2
0
rMuV
rMuV-WA1
rMuV-B.1.1.7
rMuV-B.1.351
rMuV-B.1.617.2

Fig.S3. Characterization of rMuV-JL2 expressing preS-6P of SARS-CoV-2 WA1 and variants of concern. (A) Diagram of insertion of preS-6P into the MuV-JL2 genome. The *preS-6P* of SARS-CoV-2 WA1 and VoCs is inserted at the P-M gene junction in the MuV-JL2 genome. A diagram of the negative-sense RNA genome of MuV-JL2 encoding 3' leader sequence, N, P, M, F, SH, HN, L, and 5' trailer sequence is shown. **(B) Plaque morphology of recombinant MuV-JL2**. Vero CCL81 cells were used for plaque assay. Plaques were developed after 4 days of incubation at 37ºC. **(C) Western blot analysis of preS-6P expression by MuV-JL2**. Vero CCL81 cells in a 12-well-plate were infected by each recombinant virus at an MOI of 0.1. At 72 and 96h, 10 µl of cell culture supernatant (from a total of 1 ml) and 10 µl of cell lysate (from a total of 200 µl) were analyzed by Western blot using an antibody-specific for SARS-CoV-2 S. **(D) Viral titer of each recombinant virus**. Confluent Vero CCL81 in 12-well-plates were infected with each rMuV at an MOI of 0.1. When maximum cytopathic effects (CPEs) were observed, cell culture supernatant and lysate were collected for virus titration by plaque assay.

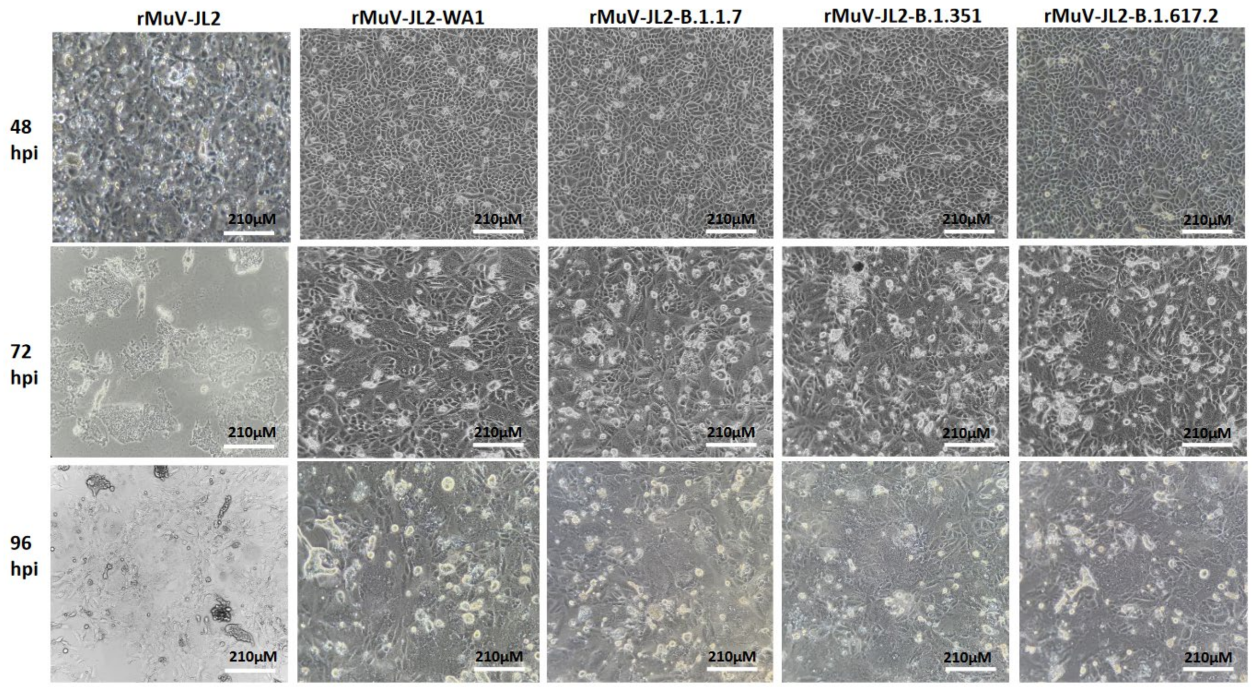

Fig.S4. Dynamics of cytopathic effects in Vero CCL81 infected by each recombinant MuV-JL2. Confluent Vero CCL81 in 12-well-plates were infected with each rMuV-JL2 at an MOI of 0.1. At 48, 72, and 96 h post-infection, representative images of cytopathic effects **(**CPE) in cells infected by each virus are shown.

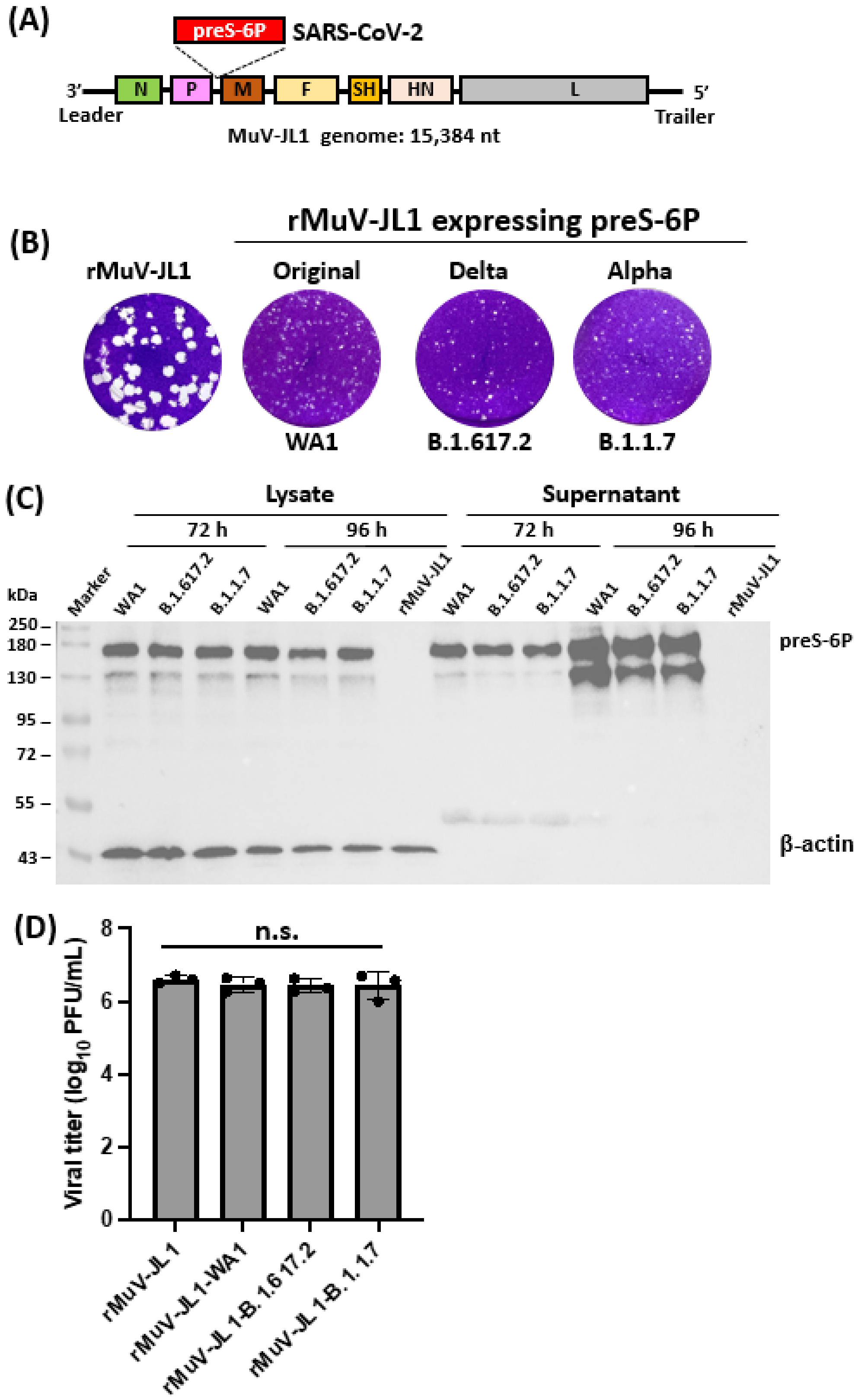
(A)
preS-6P
SARS-CoV-2
3'
N
P
M
F
SH
HN
L
5'
Leader
Trailer
MuV-JL1 genome: 15,384 nt
(B)
rMuV-JL1 expressing preS-6P
rMuV-JL1
Original
Delta
Alpha
WA1
B.1.617.2
B.1.1.7
(C)
Lysate
Supernatant
72 h
96 h
72 h
96 h
kDa
Marker
WA1
B.1.617.2
B.1.1.7
WA1
B.1.617.2
B.1.1.7
rMuV-JL1
WA1
B.1.617.2
B.1.1.7
WA1
B.1.617.2
B.1.1.7
rMuV-JL1
250
180
130
95
72
55
43
preS-6P
β-actin
(D)
n.s.
Viral titer (log10 PFU/mL)
8
6
4
2
0
rMuV-JL1
rMuV-JL1-WA1
rMuV-JL1-B.1.617.2
rMuV-JL1-B.1.1.7

Fig.S5. Characterization of rMuV-JL1 expressing preS-6P of SARS-CoV-2 WA1 and variants of concern. (A) Diagram of insertion of preS-6P into the MuV-JL1 genome. The *preS-6P* of SARS-CoV-2 WA1 and VoCs is inserted at the P-M gene junction in the MuV-JL1 genome. A diagram of the negative-sense RNA genome of MuV-JL1 encoding 3' leader sequence, N, P, M, F, SH, HN, L, and 5' trailer sequence is shown. **(B) Plaque morphology of recombinant MuV-JL1**. Vero CCL81 cells were used for plaque assay. Plaques were developed after 4 days of incubation at 37ºC. **(C) Western blot analysis of preS-6P expression by MuV-JL1**. Vero CCL81 cells in 12-well-plates were infected by each recombinant virus at an MOI of 0.1. At 72 and 96h, 10 µl of cell culture supernatant (from a total of 1 ml) and 10 µl of cell lysate (from a total of 200 µl) were analyzed by Western blot using an antibody-specific for SARS-CoV-2 S. **(D) Viral titer of each recombinant virus**. Confluent Vero CCL81 in 12-well-plates were infected with each rMuV at an MOI of 0.1. When maximum cytopathic effects (CPE) was observed, cell culture supernatant and lysate were collected for virus titration by plaque assay.

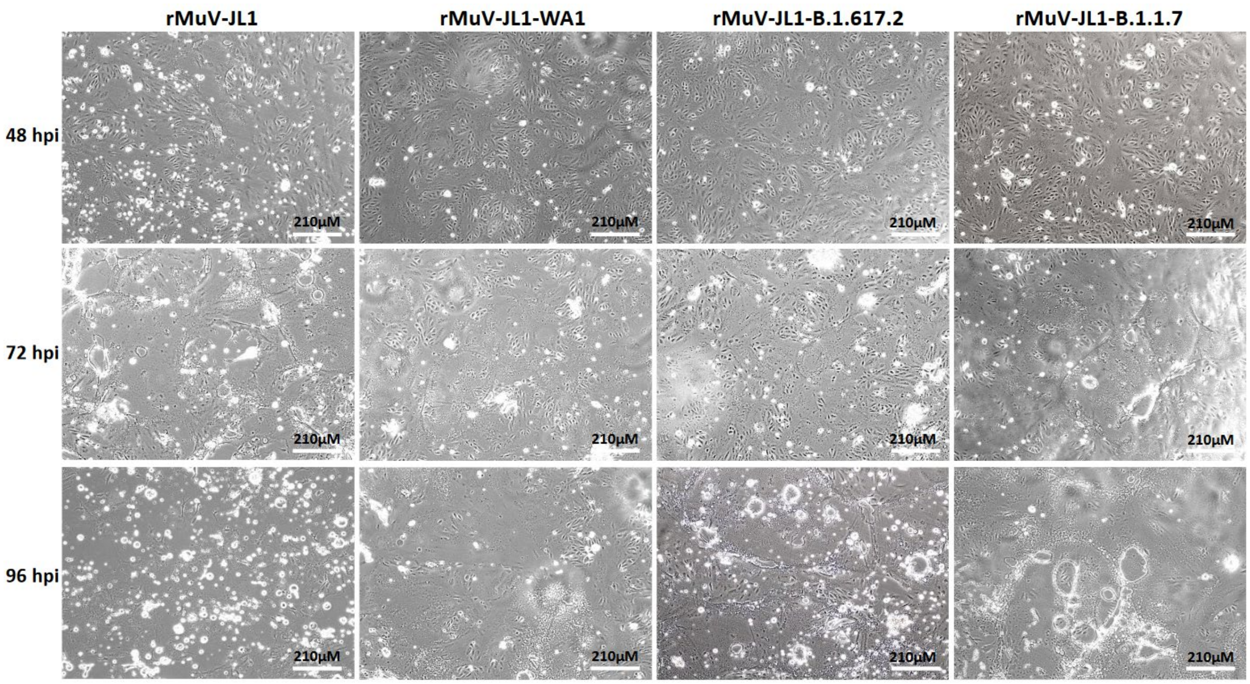

Fig.S6. Dynamics of cytopathic effects in Vero CCL81 infected by each recombinant MuV-JL1. Confluent Vero CCL81 in 12-well-plates were infected with each rMuV-JL1 at an MOI of 0.1. At 48, 72, and 96 h post-infection, representative images of cytopathic effects **(**CPE) in cells infected by each virus are shown.

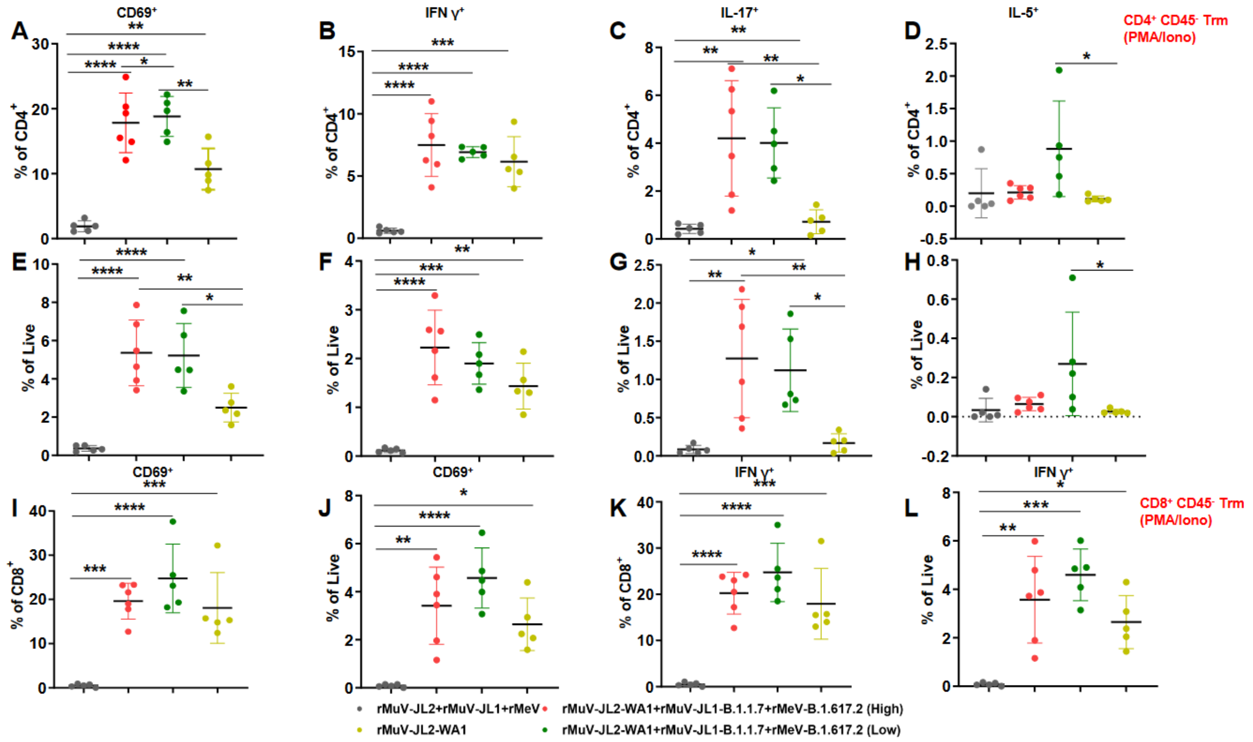

Fig.S7. Intranasal immunization of trivalent vaccine induces superior resident memory T cells to the monovalent immunization after stimulation with PMA/lono. Lung T cell suspensions from Fig.4 were stimulated with myristate acetate (PMA)/ionomycin (Iono). **(A-D)** show the percentage of CD4$^+$of S-specific CD4$^+$CD69$^+$ **(A)**, IFN-γ **(B)**, IL-17 **(C)**, and IL-5 **(D)** CD4$^+$ T cells. **(E-H)** show the percentage of live cells of S specific CD4$^+$CD69$^+$ **(E)**, IFN-γ **(F)**, IL-17 **(G)**, and IL-5 **(H)** CD4+ T cells. **(I and J)** show the percentage of CD8$^+$ **(I)** and live cells **(J)** of S-specific CD8$^+$CD69$^+$ cells. **(K and L)** show the percentage of CD8$^+$ **(K)** and live cells **(L)** of S-specific IFN-γ CD8+ T cells. In panel A, the *P* value for TVC-IV (High), TVC-IV (Low), and rMuV-JL2-WA1 vs MMM vector is ****P=2.15×10^{-6}, ****P =1.717×10^{-6}, and **P=0.0029, respectively; TVC-IV (High) vs rMuV-JL2-WA1 is *P=0.0117; and TVC-IV (Low) vs rMuV-JL2-WA1 is **P=0.006. In panel B, the *P* value for TVC-IV (High), TVC-IV (Low), and rMuV-JL2-WA1 vs MMM vector is ****P=2.041×10^{-5}, ****P =9.779×10^{-5}, and ***P=0.0004, respectively.

In panel C, the *P* value for TVC-IV (High) and TVC-IV (Low) vs MMM vector is $^{**}P=0.0036$ and $^{**}P=0.0079$, respectively; TVC-IV (High) and TVC-IV (Low) vs rMuV-JL2-WA1 is $^{**}P=0.007$ and $^{*}P=0.0149$ respectively. In panel D, the *P* value for TVC-IV (Low) vs rMuV-JL2-WA1 is $^{*}P=0.0357$. In panel E, the *P* value for TVC-IV (High) and TVC-IV (Low) vs MMM vector is $^{****}P=3.657\times10^{-5}$ and $^{****}P=8.489\times10^{-5}$, respectively; TVC-IV (High) and TVC-IV (Low) vs rMuV-JL2-WA1 is $^{**}P=0.0095$ and $^{*}P=0.0184$, respectively. In panel F, the *P* value for TVC-IV (High), TVC-IV (Low), and rMuV-JL2-WA1 vs MMM vector is $^{****}P<0.0001$, $^{***}P=0.0002$, and $^{**}P=0.0042$, respectively. In panel G, the *P* value for TVC-IV (High) and TVC-IV (Low) vs MMM vector is $^{**}P=0.0053$ and $^{*}P=0.0206$ respectively; TVC-IV (High) and TVC-IV (Low) vs rMuV-JL2-WA1 is $^{*}P=0.0095$ and $^{**}P=0.0353$, respectively. In panel H, the *P* value for TVC-IV (Low) vs rMuV-JL2-WA1 is $^{*}P=0.045$. In panel I, the *P* value for TVC-IV (High), TVC-IV (Low), and rMuV-JL2-WA1 vs MMM vector is $^{***}P=0.0003$, $^{****}P=2.64\times10^{-5}$, and $^{***}P=9.507\times10^{-4}$, respectively. In panel J, the *P* value for TVC-IV (High), TVC-IV (Low), and rMuV-JL2-WA1 vs MMM vector is $^{**}P=0.0012$, $^{***}P=8.1\times10^{-5}$, and $^{*}P=0.0159$, respectively. In panel K, the *P* value for TVC-IV (High), TVC-IV (Low), and rMuV-JL2-WA1 vs MMM vector is $^{****}P=7.377\times10^{-5}$, $^{****}P=1.0027\times10^{-5}$, and $^{***}P=0.0005$, respectively. In panel L, the *P* value for TVC-IV (High), TVC-IV (Low), and rMuV-JL2-WA1 vs MMM vector is $^{**}P=0.001042$, $^{***}P=0.000106$, and $^{*}P=0.0192$, respectively. One-way ANOVA with Tukey's multiple comparisons was used to detect differences among groups ($^{*}P<0.05$; $^{**}P<0.01$; $^{***}P<0.001$; $^{****}P<0.0001$).

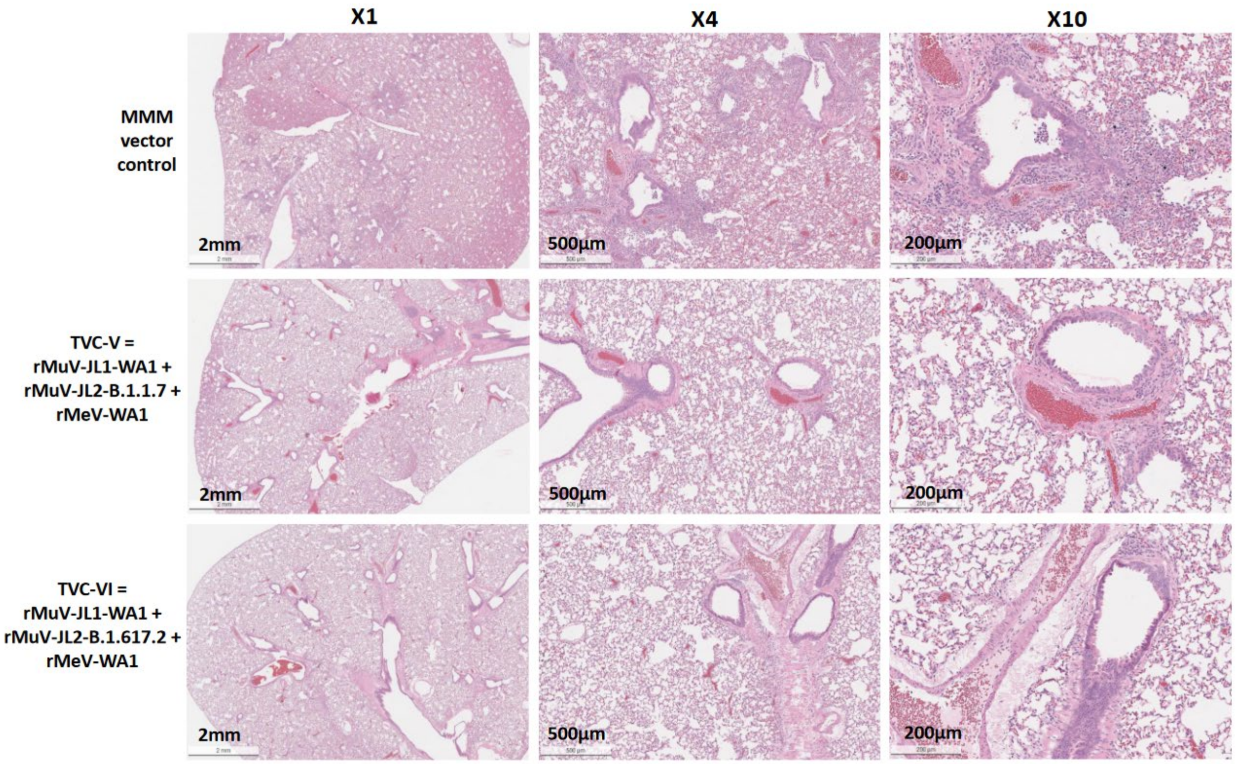

Fig.S8. Trivalent vaccine protects against lung pathology after challenge with SARS-CoV-2 WA1. Hamsters were euthanized at day 4 after challenge with SARS-CoV-2 WA1. A representative lung histologic image with 1×, 4×, and 10× magnification from each group is shown. Severe histologic changes are identified in the MMM vector control group whereas mild pathologic changes are observed in the trivalent vaccine groups. Scale bars are indicated at the left corner of each image.

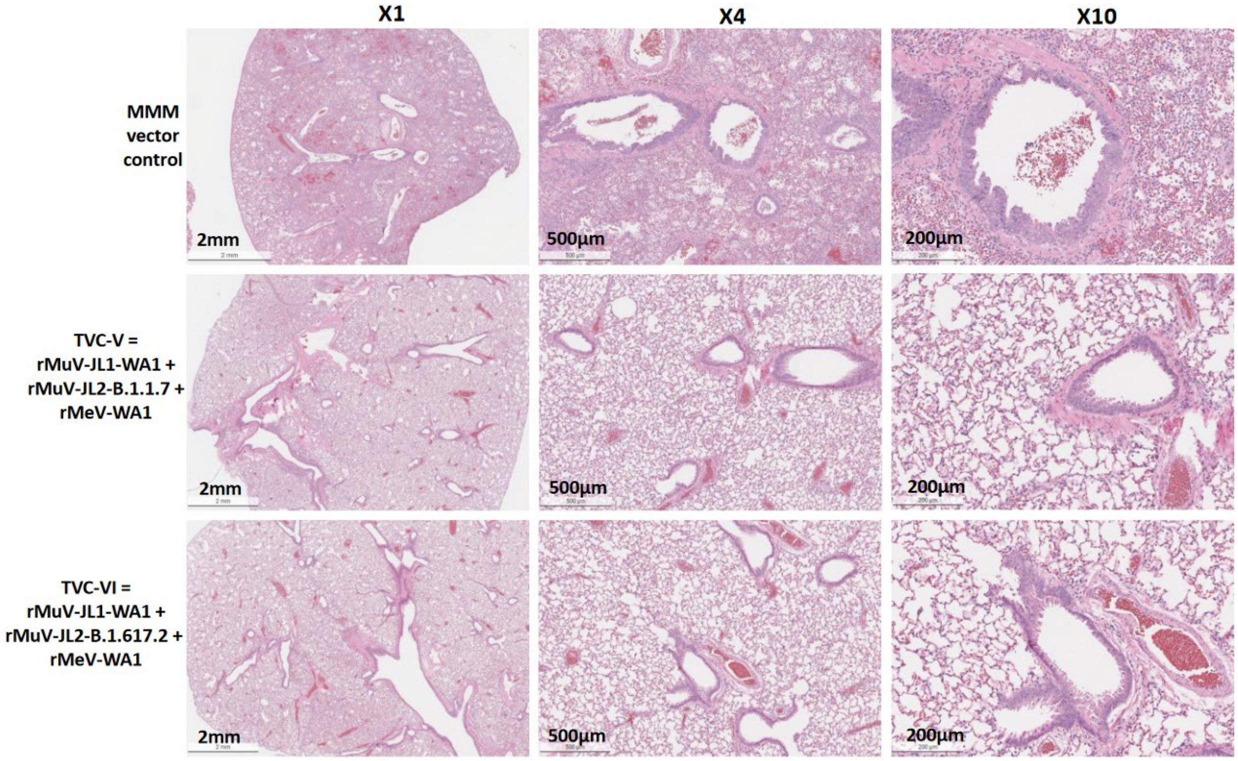

Fig.S9. Trivalent vaccine protects against lung pathology after challenge with SARS-CoV-2 B.1.617.2 VoC. Hamsters were euthanized at day 4 after challenge with SARS-CoV-2 B.1.617.2 VoC. A representative lung histologic image with 1×, 4×, and 10× magnification from each group is shown. Severe histologic changes are identified in the MMM vector control group whereas mild pathologic changes are observed in the trivalent vaccine groups. Scale bars are indicated at the left corner of each image.

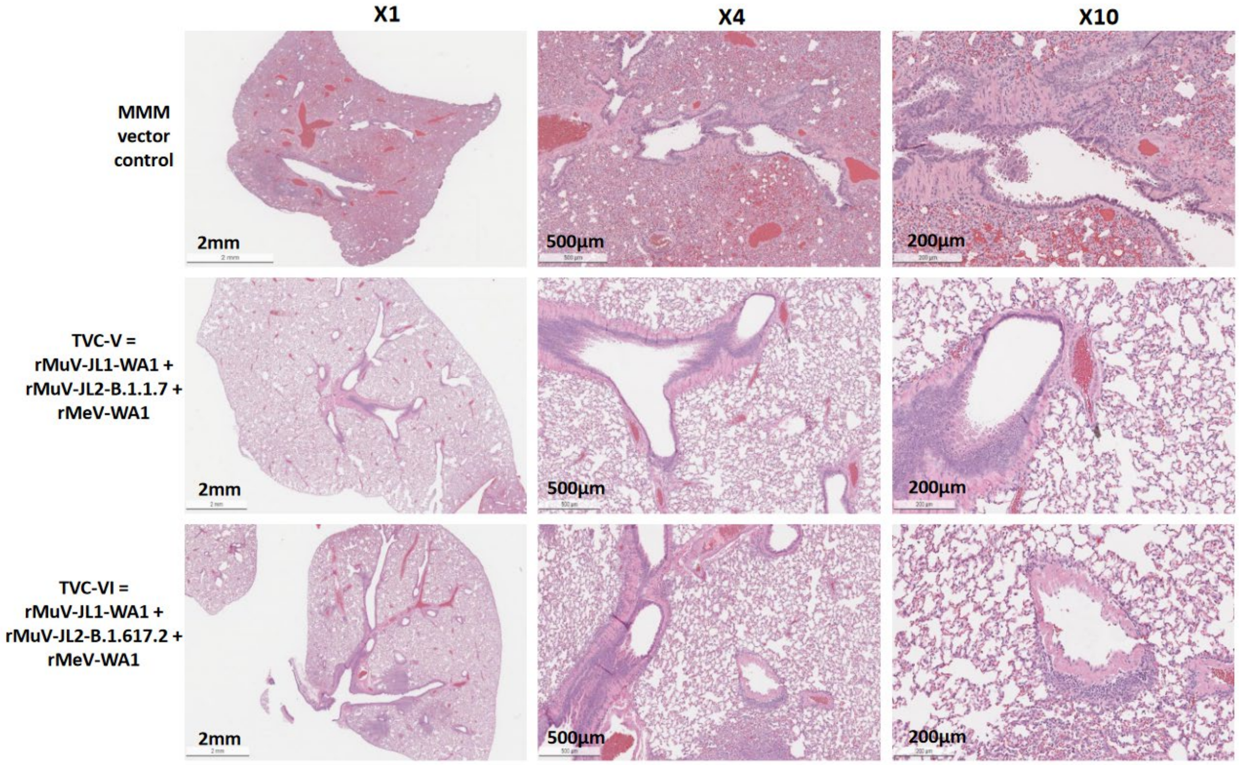

Fig.S10. Trivalent vaccine protects against lung pathology after challenge with Omicron BA.1. Hamsters were euthanized at day 3 after challenge with SARS-CoV-2 Omicron BA.1. A representative lung histologic image with 1×, 4×, and 10× magnification from each group is shown. Severe to extremely severe histologic changes are identified in the MMM vector control group whereas mild to moderate pathologic changes are observed in the trivalent vaccine groups. Scale bars are indicated at the left corner of each image.

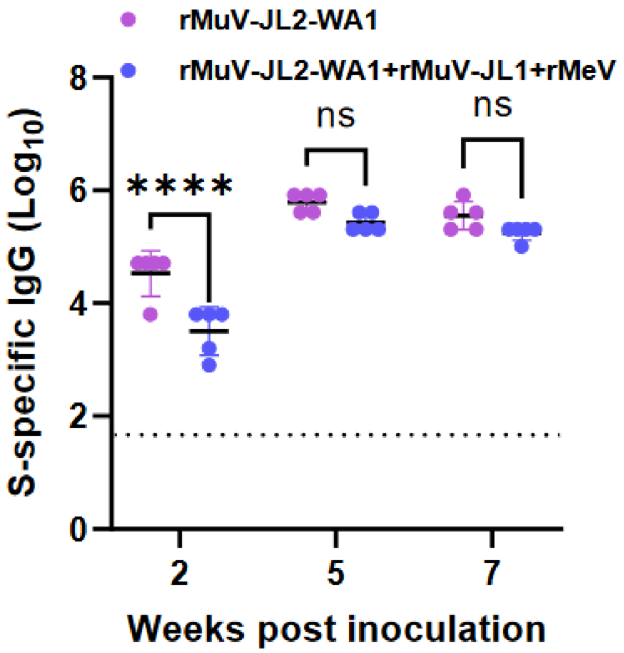

Fig.S11. The effect of the parental MeV and MuV on immune responses of rMuV-JL2-WA1. Two groups (*n*=5) of 4-week-old female hamsters were immunized with 3×10^5 PFU of MuV-JL2-WA1 (Group 1) or a mixture of 3×10^5 PFU of MuV-JL2-WA1, null MeV, and null MuV JL1 viruses (each containing 10^5 PFU) (Group 2). Three weeks later, each group was boosted with the same virus. At weeks 2, 5, and 7, serum was collected from each hamster to determine WA1-preS-6P specific IgG antibodies by ELISA. The *P* value at week 2 is ****$P= 2.0433\times10^{-5}$. Student's *t*-test was used for statistical analysis (ns > 0.05; ****$P < 0.0001$).

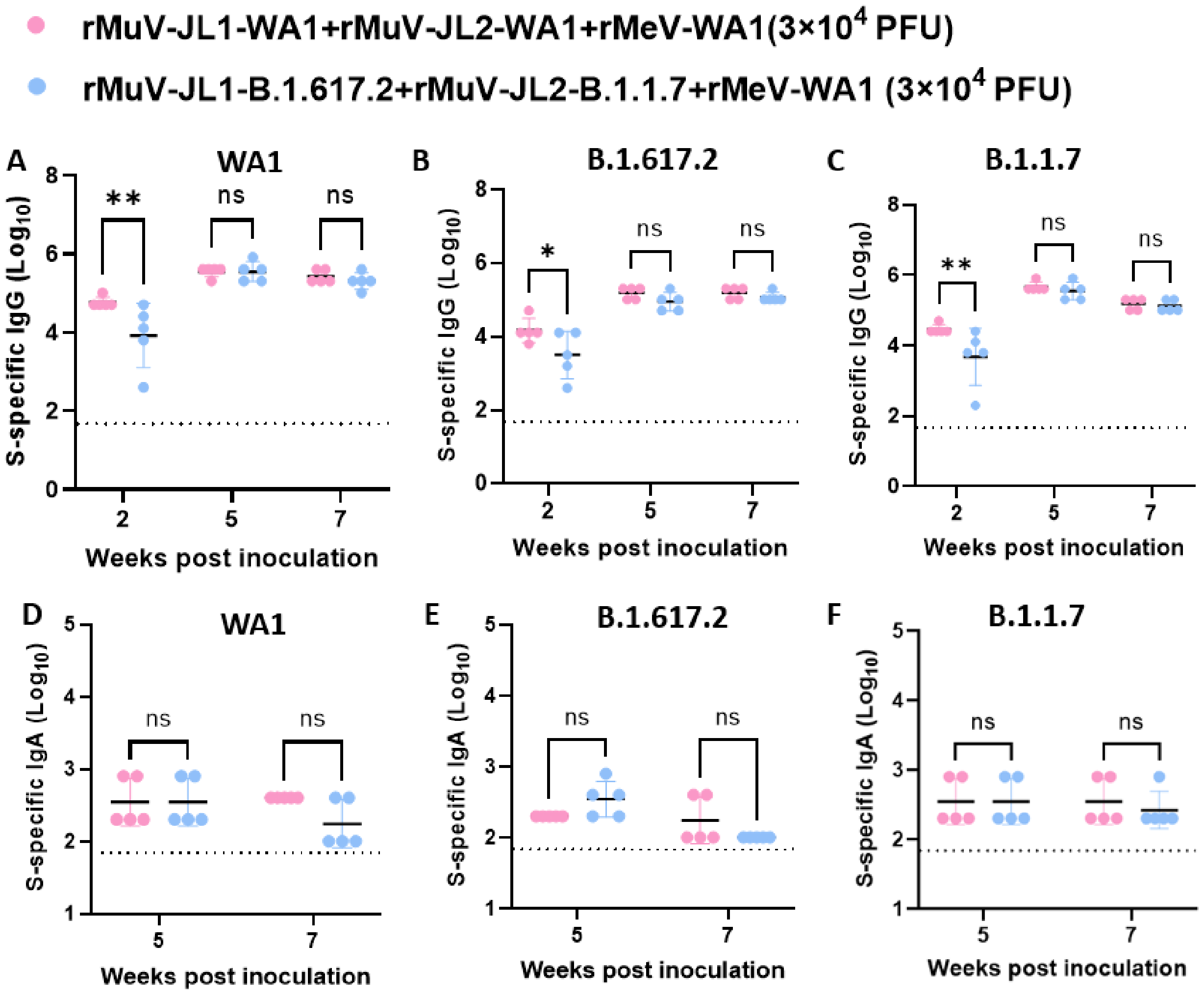

Fig.S12. Comparison of WA1-, B.1.617.2-, and B.1.1.7-specific IgG and IgA titers of TVC-VII and TVC-VIII at an immunization dose of 3×10^4 PFU. The serum IgG data at a dose of 3×10^4 PFU in **Fig.8** were re-analyzed by comparison of WA1 (A)-, B.1.617.2 (B)-, and B.1.1.7 (C)-specific IgG titer. Similarly, serum IgA data at a dose of 3×10^4 PFU in **Fig.8** were re-analyzed by comparison of WA1 (D)-, B.1.617.2 (E)-, and B.1.1.7 (F)-specific IgG titer. At weeks 5 and 7, no significant difference in VoC-specific IgG or IgA titer is observed between TVC-VII and TVC-VIII. In panel A, the *P* value at week 2 is **P=0.0046. In panel B, the *P* value at week 2 is *P=0.0122. In panel C, the *P* value at week 2 is **P=0.0078. Student's *t*-test was used for statistical analysis (ns > 0.05; *$P < 0.05$; **$P < 0.01$).

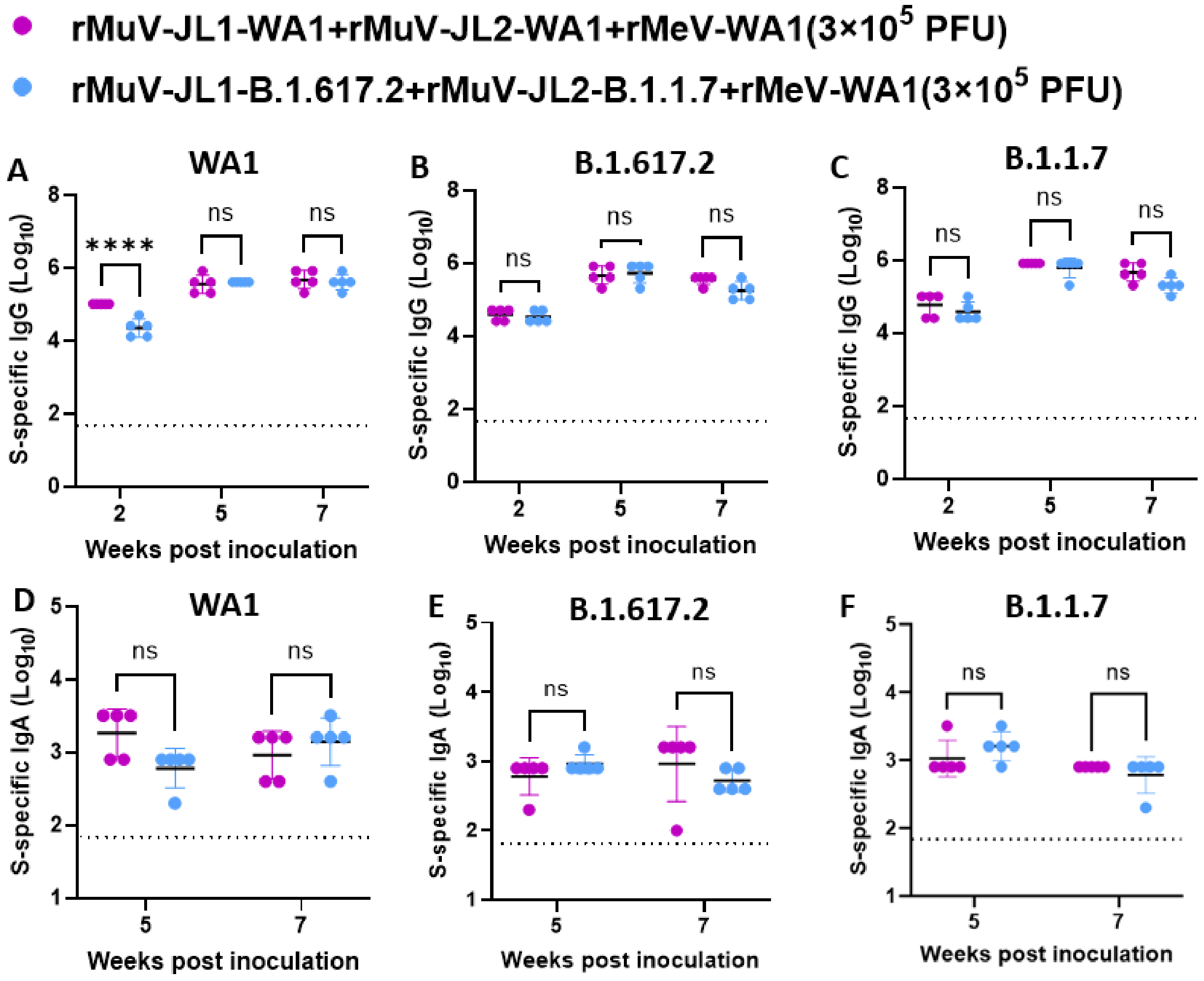

Fig.S13. Comparison of WA1-, B.1.617.2-, and B.1.1.7-specific IgG and IgA titers of TVC-VII and TVC-VIII at an immunization dose of 3×10^5 PFU. The serum IgG data at a dose of 3×10^5 PFU in **Fig.8** were re-analyzed by comparison of WA1 (A)-, B.1.617.2 (B)-, and B.1.1.7 (C)-specific IgG titer. Similarly, serum IgA data at a dose of 3×10^5 PFU in **Fig.8** were re-analyzed by comparison of WA1 (D)-, B.1.617.2 (E)-, and B.1.1.7 (F)-specific IgG titer. No significant difference in VoC-specific IgG or IgA titer is observed between TVC-VII and TVC-VIII. In panel A, the *P* value at week 2 is ****P=6.08×10^{-5}. Student's *t*-test was used for statistical analysis (ns > 0.05; ****$P < 0.0001$).

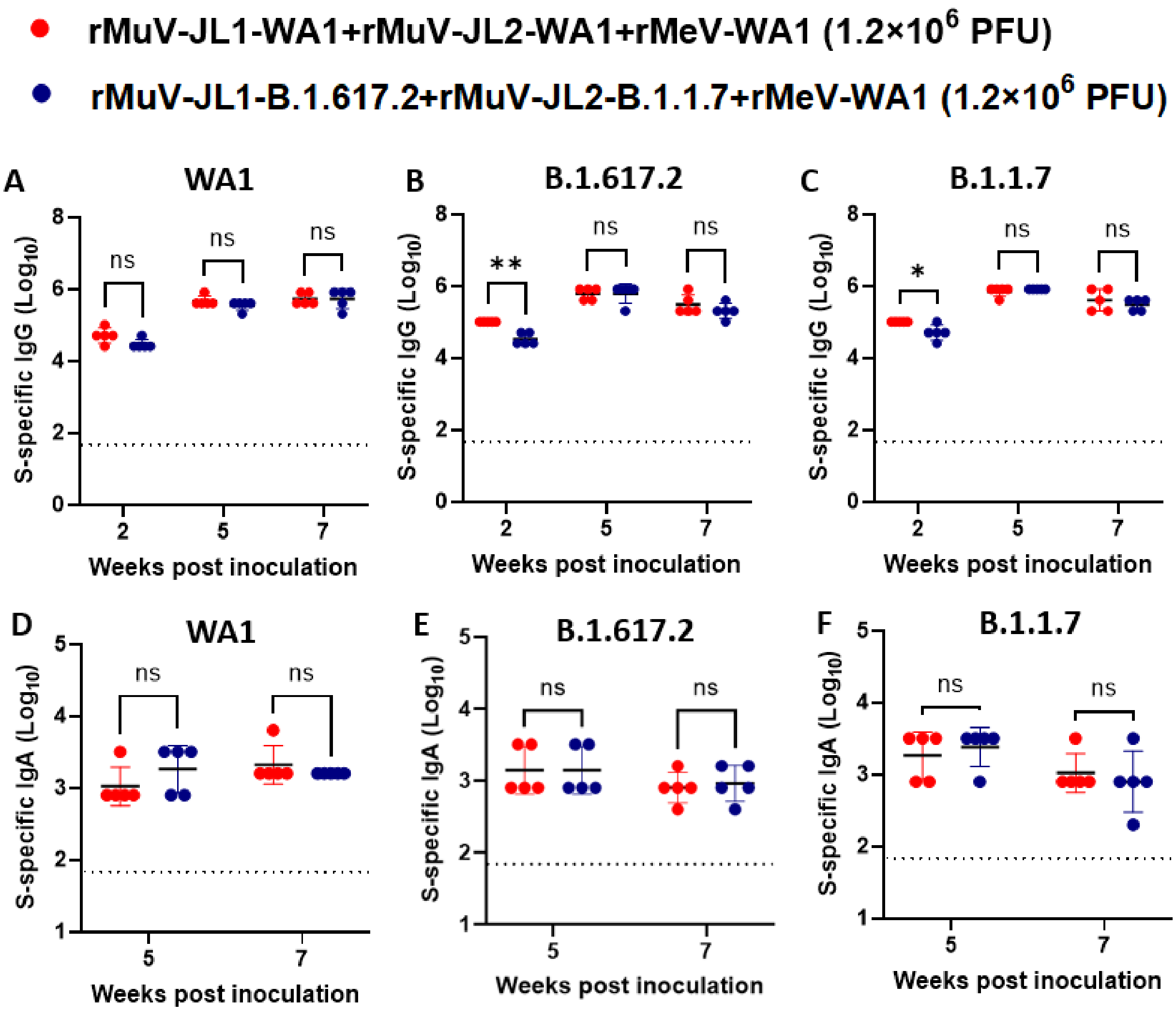

Fig.S14. Comparison of WA1-, B.1.617.2-, and B.1.1.7-specific IgG and IgA titers of TVC-VII and TVC-VIII at an immunization dose of 1.2×10^6 PFU. The serum IgG data at a dose of 1.2×10^6 PFU in **Fig.8** were re-analyzed by comparison of WA1 (A)-, B.1.617.2 (B)-, and B.1.1.7 (C)-specific IgG titer. Similarly, serum IgA data at a dose of 1.2×10^6 PFU in **Fig.8** were re-analyzed by comparison of WA1 (D)-, B.1.617.2 (E)-, and B.1.1.7 (F)-specific IgG titer. At weeks 5 and 7, no significant difference in VoC-specific IgG or IgA titer is observed between TVC-VII and TVC-VIII. In panel B, the *P* value at week 2 is ***P*=0.0028. In panel C, the *P* value at week 2 is ***P*=0.0339. Student's *t*-test was used for statistical analysis (ns > 0.05; **P* < 0.05; ***P* < 0.01).

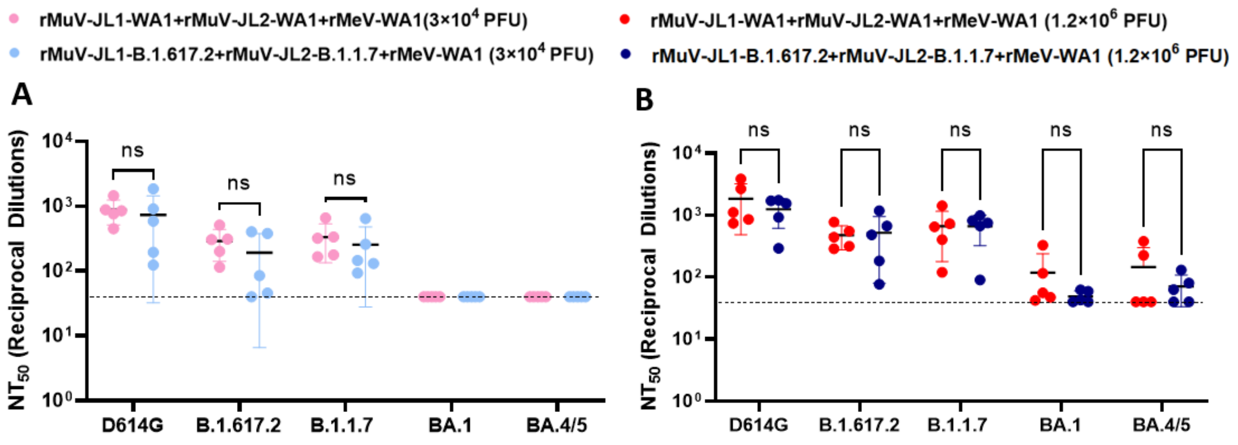

Fig.S15. Comparison of NAb between TVC-VII and TVC-VIII. The serum NAb data in Fig.8F and L were re-analyzed by comparison of WA1 (D614G)-, B.1.617.2-, B.1.1.7-, BA.1-, and BA.4/5-specific NAb titer at doses of 1.2×10^6 PFU (A) and 1.2×10^6 PFU (B). No significant difference VoC-specific NAb between TVC-VII and TVC-VIII is observed. Student's *t*-test was used for statistical analysis (ns > 0.05).

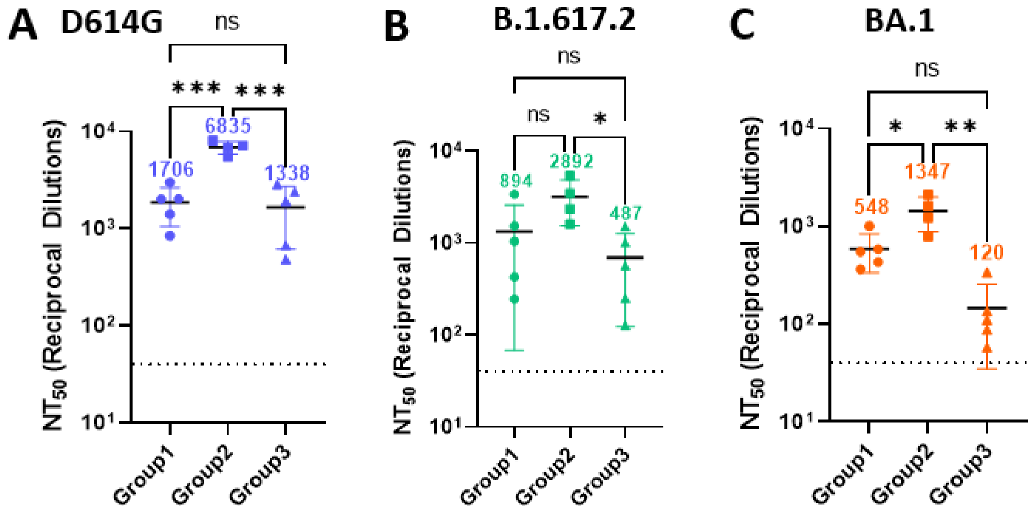

Group 1: rMuV-JL2-WA1 (1st immunization) + rMuV-JL2-WA1 (2nd immunization) + TVC-VIX (rMeV-BA.1 + rMuV-JL1-B.1.617.2 + rMuV-JL2-WA1) (3rd immunization)
Group 2: rMuV-JL2-WA1 (1st immunization) + rMuV-JL2-WA1 (2nd immunization) + monovalent rMeV-BA.1 (3rd immunization)
Group 3: rMuV-JL2-WA1 (1st immunization) + rMuV-JL2-WA1 (2nd immunization) + rMuV-JL2-WA1 (3rd immunization)

Fig.S16. The impact of immune imprinting on induction of D614G-, B.1.617.2-, and BA.1-specific NAb of a trivalent vaccine. The NAb data in Fig.10D were re-analyzed by comparison of WA1(D614G) (A), B.1.617.2 (B), and BA.1 (C) specific NAb. In panel A, the *P* value for Group 1 vs Group 2 is ***P=0.0007; Group 2 vs Group 3 is ***P=0.0005. In panel B, the *P* value for Group 2 vs Group 3 is *P=0.0279. In panel C, the *P* value for Group 1 vs Group 2 is *P=0.0163; Group 2 vs Group 3 is **P=0.0017. Data were compared using Student's *t*-test (ns > 0.05; *$P < 0.05$; **$P < 0.01$; ***$P < 0.001$).

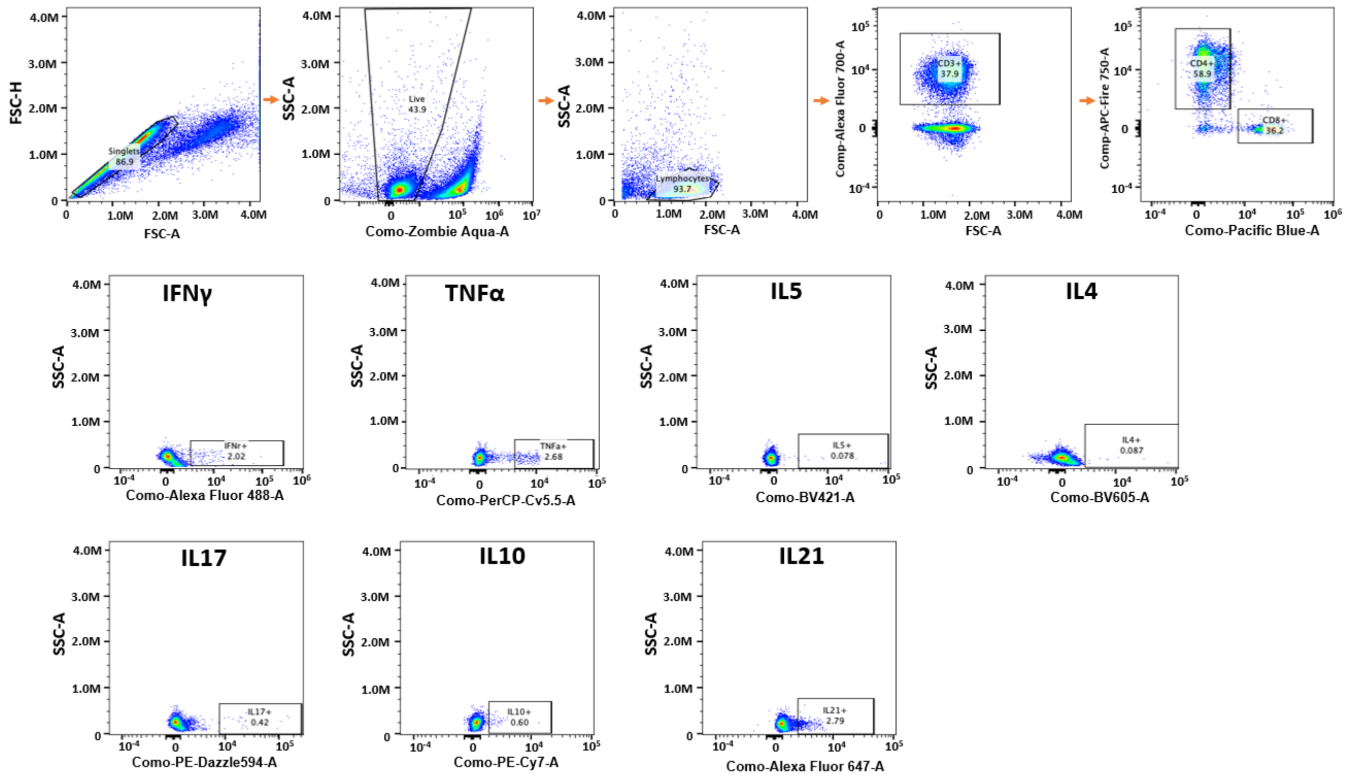

Fig.S17. Gating strategy for flow cytometry analysis of spleen T cells. The gating was set up to minimize spill-over events. The same gating strategy was applied to all samples.

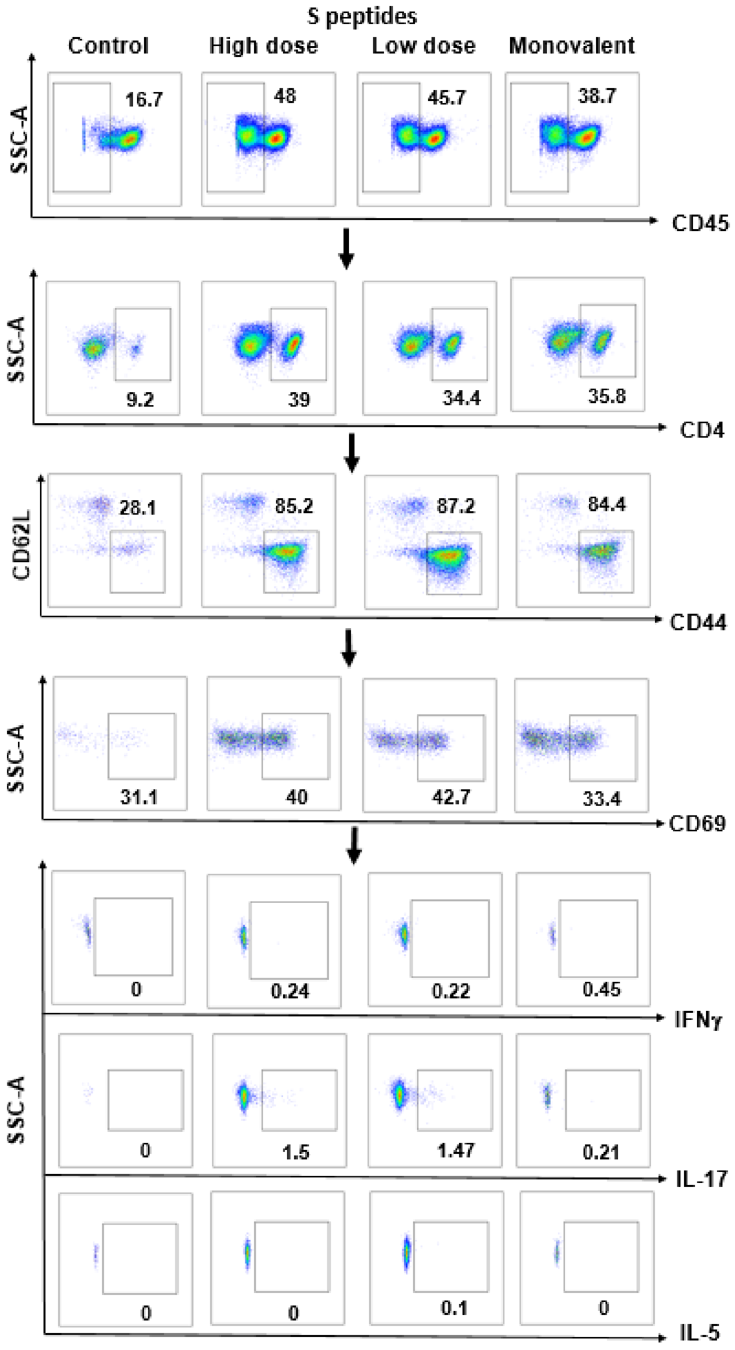

Fig.S18. Gating strategy for flow cytometry analysis of lung CD4$^+$ T cells stimulated by S peptides.

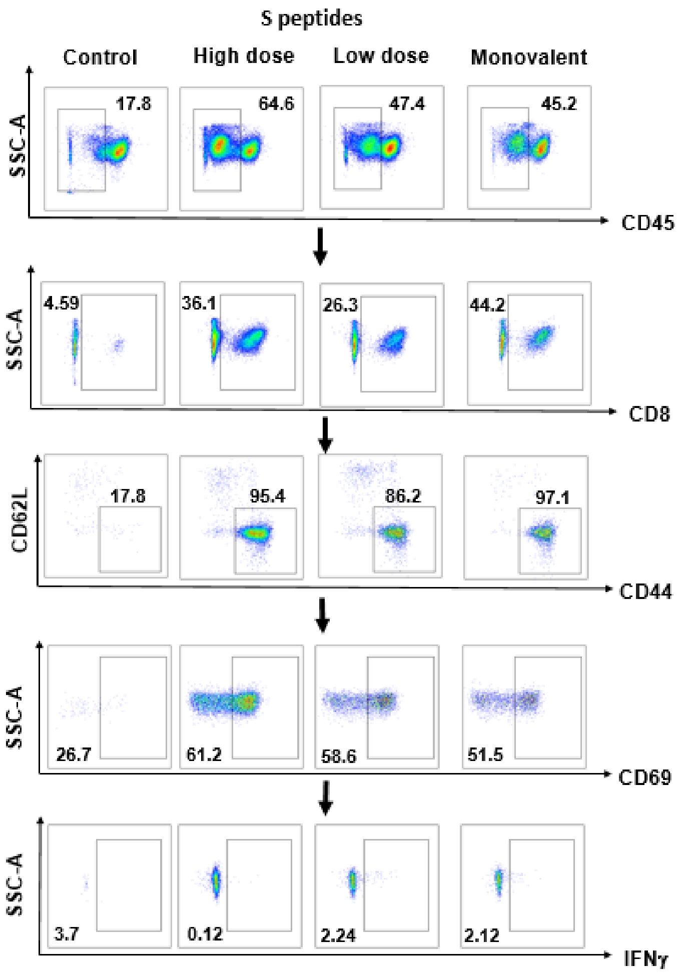

Fig.S19. Gating strategy for flow cytometry analysis of lung CD8$^+$ T cells stimulated by S peptides.

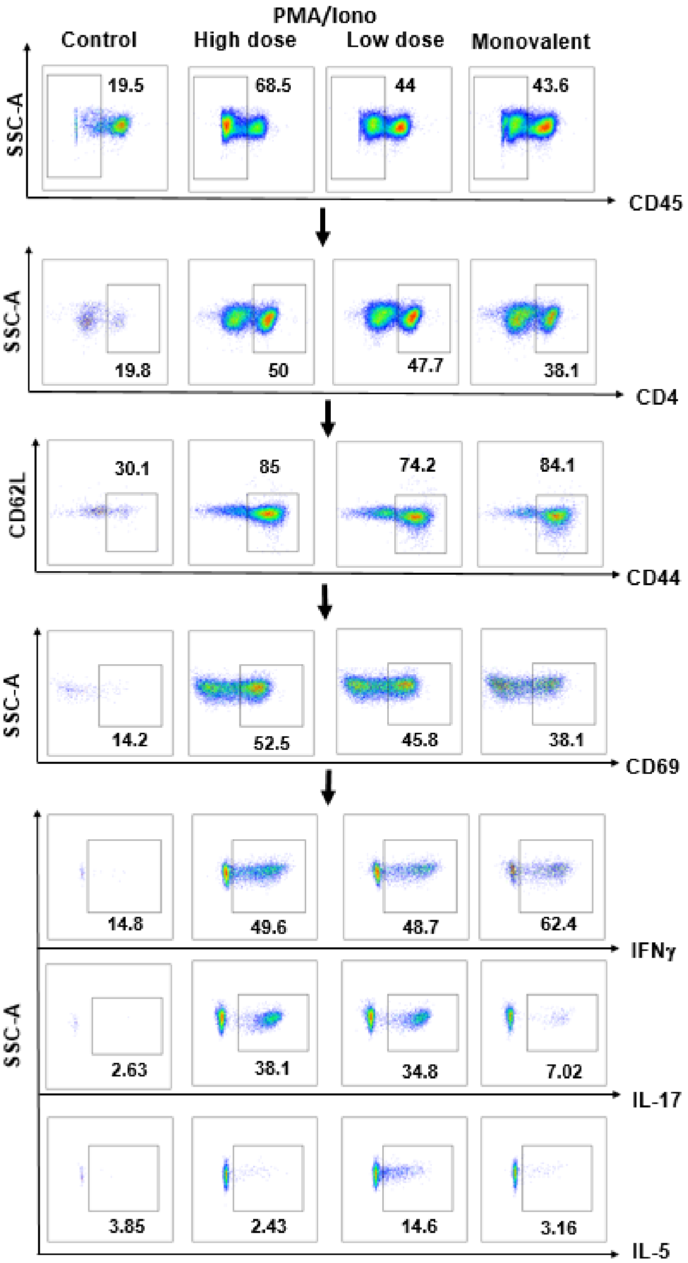

Fig.S20. Gating strategy for flow cytometry analysis of lung CD4⁺ T cells stimulated by PMA/Ionomycin.

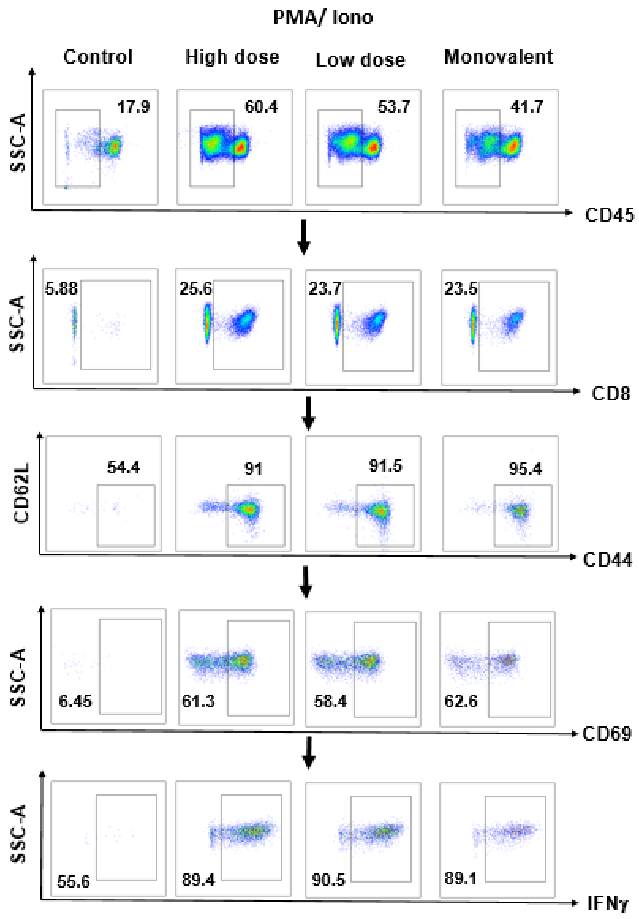

Fig.S21. Gating strategy for flow cytometry analysis of lung CD8^{+} T cells stimulated by PMA/Ionomycin.

Table S1: Recombinant viruses generated in this study

Recombinant virus	Targeted antigen	Origin
rMeV-WA1	Expresses preS-6P gene of SARS-CoV-2 WA1	Zhang et al., JMV, 2023
rMeV-B.1.351	Expresses preS-6P gene of B.1.351 VoC	This study
rMeV-B.1.1.7	Expresses preS-6P gene of B.1.1.7 VoC	This study
rMeV-B.1.617.2.	Expresses preS-6P gene of B.1.617.2.VoC	This study
rMeV-BA.1	Expresses preS-6P gene of Omicron BA.1	Xu et al., PNAS, 2023
rMuV-JL2-WA1	Expresses preS-6P gene of SARS-CoV-2 WA1	Zhang et al., PNAS, 2022
rMuV-JL2-B.1.351	Expresses preS-6P gene of B.1.351 VoC	This study
rMuV-JL2-B.1.1.7	Expresses preS-6P gene of B.1.1.7 VoC	This study
rMuV-JL2-B.1.617.2	Expresses preS-6P gene of B.1.617.2.VoC	This study
rMuV-JL1-WA1	Expresses preS-6P gene of SARS-CoV-2 WA1	This study
rMuV-JL1-B.1.1.7	Expresses preS-6P gene of B.1.1.7 VoC	This study
rMuV-JL1-B.1.617.2	Expresses preS-6P gene of B.1.617.2.VoC	Xu et al., PNAS, 2023

Table S2: Trivalent vaccine candidates tested in this study

Vaccine candidate	Composition	Rationale	Experiment
Trivalent Vaccine Candidate I (TVC-I)	rMuV-JL2-WA1, rMuV-JL2-B.1.1.7, and rMeV-B.1.351	3 viruses expressing 3 different preS-6P proteins	Animal Experiment 1
TVC-II	rMuV-JL2-WA1, rMuV-JL2-B.1.1.7, and rMeV-WA1	rMeV and rMuV-JL2 expressing WA1, and rMuV-JL2 expressing B.1.1.7	Animal Experiment 1
TVC-III	rMuV-JL1-WA1, rMuV-JL2-B.1.617.2, and rMeV-B.1.351	3 viruses expressing 3 different preS-6P proteins	Animal Experiment 2
TVC-IV	rMeV-B.1.617.2, rMuV-JL1-B.1.1.7, and rMuV-JL2-WA1	3 viruses expressing 3 different preS-6P proteins	Animal Experiment 3
TVC-V	rMuV-JL1-WA1, rMuV-JL2-B.1.1.7, and rMeV-WA1	rMeV and rMuV-JL1 expressing WA1, and rMuV-JL2 expressing B.1.1.7	Animal Experiment 4
TVC-VI	rMuV-JL1-WA1, rMuV-JL2-B.1.617.2, and rMeV-WA1	rMeV and rMuV-JL1 expressing WA1, and rMuV-JL2 expressing B.1.617.2	Animal Experiment 4
MMM vector	rMeV, rMuV-JL1, and rMuV-JL2	3 vector control	Animal Experiments 1-4
Monovalent	rMuV-JL2-WA1	Monovalent	Animal Experiments 1, 3, and 5
Monovalent	rMuV-JL2-B.1.617.2	Monovalent	Animal Experiment 4
TVC-VII	rMuV JL2-WA1 + rMuV-JL1-WA1 + rMeV-WA1	3 viruses expressing the same preS-6P of WA1	Animal Experiment 6
TVC-VIII	rMeV-WA1 + rMuV- JL1-B.1.617.2+ rMuV-JL2-B.1.1.7	3 viruses expressing 3 different spikes	Animal Experiments 6 and 7
TVC-IX	rMeV-BA.1 + rMuV-JL1-B1.617.2 + rMuV-JL2-WA1	3 viruses expressing 3 different spikes for immune imprinting experiment	Animal Experiment 8
Monovalent	rMeV-BA.1	Monovalent vaccine for immune imprinting Experiment	Animal Experiment 8

Table S3: Primers used in this study

Name	Sequences (5' to 3')
**Primers used for construction of infectious cDNA clone of MeV-SARS-CoV-2 spike**	
MeV-5end-F	ACCAAACAAAGTTGGGTAAGGATA
MeV-N-R	GGTAGGCGGATGTTGTTCTG
MeV-P-F	CTTCTAGACTAGGTGCGA
MeV-P (WA1/B.1.617.2/B.1.351/B.1.1.7/ BA.1)-R	GCAGGAGCACCAGGAACACGAACATTATACGCGTTGATGGGCT GGCG
MeV-M (B.1.351/B.1.1.7/BA.1)-F	GTACTGCTGAGCACATTCCTGGGCTGATACAACCTAAATCCATT ATAAA
MeV-M (WA1/B.1.617.2)-F	GTACTGCTGAGCACATTCCTGGGCTAATGATACAACCTAAATCC ATTATAAA
MeV-M-R	CATGAATATGGCAGAGACGT
MeV-F-F	CCCGACGACACTCAACTCCC
MeV-H-R	ACGTTTTTCTTAATTCTGATGTCTAT
MeV-L1-F	ACATCAGGCATACCCACTA
MeV-L1-R	CCCACATATGGCTTCTTAG
MeV-L2-F	GACAAAGAGTCATGTTCAGTG
MeV-3end-R	CAGACAAAGCTGGGAATAG
pYES2-F	GCAATATATTAAAGAAAACTTTGAAAATACG
MeV-5end-R	GCACTAGAAGATGATCATTGATTGAAC
preS-6P-(WA1/B.1.617.2/B.1.351/B.1.1.7/ BA.1)-F	CGCCAGCCCATCAACGCGTATAATGTTCGTGTTCCTGGTGCTCC TGC
preS-6P-(WA1/B.1.617.2)-R	TTTATAATGGATTTAGGTTGTATCATTAGCCCAGGAATGTGCTCA GCAGTAC
preS-6P-(B.1.351/B.1.1.7/BA.1)-R	TTTATAATGGATTTAGGTTGTATCAGCCCAGGAATGTGCTCAGC AGTAC
**Primers used for construction of infectious cDNA clone of MuV-JL2-SARS-CoV-2 spike**	
MuV-JL2-NP-F	ACC AAG GGG AAA ATG AAG ATG
MuV-JL2-NP-(WA.1/ B.1.351/B.1.1.7/B.1.617.2)-R	GCAGGAGCACCAGGAACACGAACATGCCTGTGATCGTGGACC TAATTCTTTCCGGGCCTAATTTTTTATTTAATAGGGAATCATTGT
MuV-JL2-M-F-(WA.1/B.1.617.2) -F	GTACTGCTGAGCACATTCCTGGGCTGAGAGAGCCACAATGATT CCCTATTAAATAAAAAATAAGCACGAACACAAGTCGAATCCAA C
MuV-JL2-M-F-(B.1.351/B.1.1.7) -F	GTACTGCTGAGCACATTCCTGGGCTGAAGCCACAATGATTCCC TATTAAATAAAAAATAAGCACGAACACAAGTCGAATCCAAC
MuV-JL2-M-F-R	TAAGGGAGGTTGGATTGCCG
MuV-JL2-SH-HN-F	CTA GGG TCG TAA CGT CTC
MuV-JL2-SH-HN-R	TAA GAA ATG AGA CAC GCC
MuV-JL2-L1-F	GAG TTG TAG TGA ATG TAG TAGG

MuV-JL2-L1-R	GTA TTC TAT TAC CGT ATT CAGC
MuV-JL2-L2-F	AGA CCA CTG TCA GCA AAG
MuV-JL2-L2-R	ACC AAG GGG AGA AAG TAG
pYES-MuV-JL2-F	CATCAGTATTAAATCTAGGAGATCC
pYES-MuV-JL2-R	CTGTTTCTTACACTATTTGTTCTACCA
preS-6P-(WA1/B.1.351/B.1.1.7/ B.1.617.2)-F	ACAATGATTCCCTATTAAATAAAAAATTAGGCCCGGAAAGAATT AGGTCCACGATC ACAGGCATGTTCGTGTTCCTGGTGCTCCTGC
preS-6P-(WA1/B.1.617.2)-R	GTTGGATTCGACTTGTGTTCGTGCTTATTTTTATTTAATAGGGA ATCATTGTGGCTCTCT CAGCCCAGGAATGTGCTCAGCAGTAC
preS-6P-(B.1.351/B.1.1.7)-R	GTTGGATTCGACTTGTGTTCGTGCTTATTTTTATTTAATAGGGA ATCATTGTGGCTTCAGCCCAGGAATGTGCTCAGCAGTAC
**Primers used for construction of infectious cDNA clone of MuV-JL1-SARS-CoV-2 spike**	
MuV-JL1-NP-F	ACCAAGGGGAGAATGAATATG
MuV-JL1-NP-(WA1/B.1.1.7/ B.1.617.2)-R	GCAGGAGCACCAGGAACACGAACATGCCTGTGATCGTGGACC TAATTCTTTCCGGGCCTAATTTTTTATTTAATAGGGAATCATTGT
MuV-JL1-M-F-(WA1/ B.1.617.2) -F	GTACTGCTGAGCACATTCCTGGGCTGAGAGAGCCACAATGATT CCCTATTAAATAAAAAATAAGCACGAACACAAGTCAAATCC
MuV-JL1-M-F-(B.1.1.7)-F	GTACTGCTGAGCACATTCCTGGGCTGAAGCCACAATGATTCCC TATTAAATAAAAAATAAGCACGAACACAAGTCAAATCC
MuV-JL1-M-F-F	CACCGAGGATGCTCTGAACGAT
MuV-JL1-M-F-R	TAAGGGAGGTTGGATTGCCG
MuV-JL1-SH-HN-F	CTAGGGTCGTAACGTCTC
MuV-JL1-SH-HN-R	TAAGAAATGAGACACGCC
MuV-JL1-L1-F	GAGTTGTAGTGAATGTAGCAGG
MuV-JL1-L1-R	ATATTCTATTACTGTATTCAGC
MuV-JL1-L2-F	AGGCCACTGTCAGCAAAG
MuV-JL1-L2-R	ACCAAGGGGAGAAAGTAA
pYES-MuV-JL1-R	CTGTTTCTTACACTATTTGTTCTACCA
pYES-MuV-JL1-F	CATCAGTATTAAATCTAGGAGATCC
preS-6P- (WA1/B.1.1.7/B.1.617.2)-F	ACAATGATTCCCTATTAAATAAAAAATTAGGCCCGGAAAGAATT AGGTCCACGATCACAGGCATGTTCGTGTTCCTGGTGCTCCTGC
preS-6P-(WA1/B.1.617.2)-R	GGATTTGACTTGTGTTCGTGCTTATTTTTATTTAATAGGGAATC ATTGTGGCTCTCTCAGCCCAGGAATGTGCTCAGCAGTAC
preS-6P-(B.1.1.7)-R	GGATTTGACTTGTGTTCGTGCTTATTTTTATTTAATAGGGAATC ATTGTGGCTTCAGCCCAGGAATGTGCTCAGCAGTAC

Supplementary Discussion

Urgent need to develop an intranasal SARS-CoV-2 vaccine. The mucosal surface and mucosal immune mechanisms provide barrier against potentially dangerous pathogens. Therefore, enhancing mucosal immunity through vaccines holds significant promise for reducing the burden of viral disease, particularly respiratory virus infections[1]. Although the current approved vaccines (mRNA-based vaccine, adenovirus-based vaccines, and subunit vaccines) induce strong serum NAbs, they do not induce sufficient mucosal immune responses[2-4]. These vaccines are effective against severe diseases and hospitalization but are not sufficient to prevent disease transmission and virus infection. Injectable mRNA or adenovirus-based vaccines do not appear to induce S-specific $CD4^+$ and $CD8^+$ T cells in the airways of immunized macaques[5]. An adenovirus-based COVID-19 vaccine (ChAdOx1 nCoV-19, University of Oxford / AstraZeneca) failed to induce mucosal immune responses in human clinical trials even though it has been administered via the intranasal route[6]. Thus, the ideal next generation SARS-CoV-2 vaccines should generate robust systemic and mucosal immune responses.

Currently, several mucosal vaccine candidates including chimpanzee adenovirus-vectored SARS-CoV-2 vaccine candidates (ChAd-SARS-CoV-2-S)[7], adenovirus type 5 (Ad5)-based S vaccine, bivalent Ad26 based SARS-CoV-2 vaccine[8], a murine pneumonia virus vectored vaccine (MPV/S-2P) [9], parainfluenza virus type 3 (PIV3) vectored (B/HPIV3/S-6P) [10], and Newcastle disease virus (NDV) expresses a stable version of the spike protein (NDV-HXP-S) [11] have been reported. Similar to our observations, intranasal delivery of these vaccines in rodents, nonhuman primate, or human clinical trial induced strong mucosal and systemic immune responses and superior protection compared to the conventional immunization routes (e.g. I.M. and S.C.).

The roles of T cell in protection against SARS-CoV-2 infection. Numerous studies have shown that T cell immune response play a critical roles in protection against SARS-CoV-2 infection[12,13]. Antibodies derived from WA1 spike are not effective in neutralizing Omicron variants and subvariants. However, the conserved T cell epitopes in spikes may provide cross-protection against Omicron variant. For example, it was found that cynomolgus macaques immunized with the BNT162b2 and Ad.26.COV2.S had markedly lower Omicron-specific NAbs than WA1-specific NAbs, whereas Omicron-specific T cell responses were comparable to WA1/2020-specific T cell responses, indicating substantial cross-reactivity of cellular immune responses against SARS-CoV-2 BA1[14]. Moreover, it was reported that BNT162b2 and Ad.26.COV2.S provided 70% and 85% protection, respectively, against hospitalization with Omicron in South Africa, despite the absence of Omicron-specific NAbs[15]. In addition, Gao et et., showed SARS-CoV-2 S-specific $CD4^+$ and $CD8^+$ T cells elicited by BNT162b2 vaccination are also effective against B.1.1.529 [16]. These data suggest that immune parameters other than NAb responses likely contribute to protection against severe disease.

Comparison of spleen T cell response with other studies. We also found that both *i.n.* and *s.c.* immunization induced $CD4^+$ and $CD8^+$ T cells in splenocytes with a *predominant Th1/17* response. However, within the Th1 cytokines, percent of IFN-γ^+ cells was significantly less than percent of TNF-α^+ cells (**Fig.6A, B, H, and I**). Similar to our study, Hörner et al., observed lower number of CD4+ IFN-γ^+ cells than $CD4^+$TNF-α^+ cells in IFNAR$^{-/-}$-CD46Ge mice intraperitoneally immunized with 1×10^5 $TCID_{50}$ of MeV_{vac2}-SARS2-S(H) (MeV expressing full-length spike)[17]. In contrast, Frantz et al., observed similar number of IFN-γ^+ cells and TNF-α^+ cells in IFNAR$^{-/-}$ mice

immunized intraperitoneally with 1×10^5 $TCID_{50}$ of MV-ATU2-SF-2P-dER (MeV expressing preS-2P of SARS-CoV-2)[18]. The reasons for these discrepancies remain unknown, probably due to the difference in antigen compositions, animal models, doses, and immunization routes. However, in all cases, we and others consistently observed a predominant Th1 response.

The immunization doses and animal models used in this study. Typically, an immunization dose of 10^5-10^7 PFU or $TCID_{50}$ was used for testing the efficacy of MeV and MuV-vectored vaccines in small animal models[17-21]. These doses used in small animal models seem higher than those (10^3-10^4 PFU) used in MMR vaccine for humans. Since human is the natural host of MeV and MuV, a low dose in MMR formulation is sufficient to induce strong immune responses. The relatively higher dose used in rodent models is likely because the homology of viral entry receptors between rodents and humans is low[19]. Nonetheless, we found that 3×10^4 PFU of trivalent vaccine is sufficient to induce a strong immune response in hamsters (**Fig.8**). In mouse experiment, a low dose (3×10^5 PFU) of trivalent vaccine included similar levels of T cell immune responses compared to the high dose (1.2×10^6 PFU) of trivalent vaccine (**Fig.5**). A dose of 3×10^5 PFU is sufficient to induce a higher level of Trms (**Fig.5**). It should be noted that neither MeV nor MuV infects immunocompetent mice efficiently. However, $IFNAR^{-/-}$ mice lacking type I interferon receptor are susceptible to infection by both MeV and MuV, and are the only mice models widely used for testing the efficacy of MeV- and MuV-based vaccines[22,23]. Despite the fact that these mice lack interferon responses, the trivalent vaccine induces robust antibody, T cell immune responses, and protection in these mice. Importantly, the immune responses and protective efficacy are verified in hamsters, the gold standard small animal model for testing SARS-CoV-2 vaccines.

Multivalent vaccine may generate broader immunity. The multivalent vaccine approach has been successful in preventing human papillomavirus virus (HPV). They protect against several HPV serotypes that can infect the genital area, as well as the mouth and throat[24]. The two main HPV vaccines currently in use are Gardasil 9 and Cervarix. Gardasil 9 protects against nine HPV types, including those that are associated with cervical, vulvar, vaginal, and anal cancers, as well as genital warts. The effectiveness of the HPV vaccines is notably high, up to 90% effective in preventing HPV-related cancers and diseases[25,26]. Using the MMR vaccine platform, trivalent or quadrivalent vaccines covering relevant circulating VoCs, updated each year, would likely provide the necessary depth and breadth of immune response for contemporary protection.

Although MeV and MuV are the respiratory viruses, they have different cell tropism and infect different cell types. The combination of MeV and MuV may provide have synergistic effects. This probably explains the observation that trivalent vaccines TVC-I and II induced higher serum IgG than rMuV-JL2-WA1 alone and that both low and high doses of TVC-IV induced higher lung Trm immune responses than rMuV-JL2-WA1 alone. Interestingly, we found that serum IgG, IgA, and NAb induced by TVC-VIII (3 viruses expressing 3 different spikes) were not significantly different from those of TVC-VII (3 viruses all expressing the same WA1 spike). These results also indicate that 3 viruses expressing WA1 spike is more immunogenic than rMuV-JL2-WA1 alone because of the synergistic effects of the vectors. Both MeV and MuV are excellent viral vectors. Insertion of foreign antigens into MeV and MuV further attenuates the vaccine viruses, thereby enhancing their safety as vaccines and as vaccine vectors.

Rapid update of MeV and MuV-based SARS-CoV-2 vaccines. It should be noted that our TVC containing preS-6P protein of WA1, Alpha, Beta, or Delta VoC are not able to induce sufficient

cross-neutralizing antibodies against Omicron BA.1 and subvariant BA.4/5. It is likely that these trivalent vaccines will not neutralize the current circulating Omicron subvariants such as the HV.1, EG.5, XBB.1.5, JN.1 and its relatives. However, because our yeast-based recombinant system and reverse genetics system are highly efficient, we can rapidly construct rMeV, rMuV-JL1, or rMuV-JL2 expressing preS-6P proteins of the current co-circulating Omicron subvariants. For example, a trivalent vaccine containing preS-6P proteins of HV.1, XBB.1.5, and JN.1 will likely induce robust and broader NAbs against the currently prevalent Omicron subvariants.

Supplementary References

1 Iwasaki, A. Exploiting Mucosal Immunity for Antiviral Vaccines. *Annu Rev Immunol* **34**, 575-608, doi:10.1146/annurev-immunol-032414-112315 (2016).

2 Tang, J. *et al.* Respiratory mucosal immunity against SARS-CoV-2 after mRNA vaccination. *Sci Immunol* **7**, eadd4853, doi:10.1126/sciimmunol.add4853 (2022).

3 Mao, T. *et al.* Unadjuvanted intranasal spike vaccine elicits protective mucosal immunity against sarbecoviruses. *Science* **378**, eabo2523, doi:10.1126/science.abo2523 (2022).

4 Nickel, O. *et al.* Evaluation of the systemic and mucosal immune response induced by COVID-19 and the BNT162b2 mRNA vaccine for SARS-CoV-2. *PLoS One* **17**, e0263861, doi:10.1371/journal.pone.0263861 (2022).

5 Hameed, S. A., Paul, S., Dellosa, G. K. Y., Jaraquemada, D. & Bello, M. B. Towards the future exploration of mucosal mRNA vaccines against emerging viral diseases; lessons from existing next-generation mucosal vaccine strategies. *NPJ Vaccines* **7**, 71, doi:10.1038/s41541-022-00485-x (2022).

6 Madhavan, M. *et al.* Tolerability and immunogenicity of an intranasally-administered adenovirus-vectored COVID-19 vaccine: An open-label partially-randomised ascending dose phase I trial. *EBioMedicine* **85**, 104298, doi:10.1016/j.ebiom.2022.104298 (2022).

7 Bricker, T. L. *et al.* A single intranasal or intramuscular immunization with chimpanzee adenovirus-vectored SARS-CoV-2 vaccine protects against pneumonia in hamsters. *Cell Rep* **36**, 109400, doi:10.1016/j.celrep.2021.109400 (2021).

8 McMahan, K. *et al.* Mucosal boosting enhances vaccine protection against SARS-CoV-2 in macaques. *Nature*, doi:10.1038/s41586-023-06951-3 (2023).

9 Buchholz, U. *et al.* Mucosal prime-boost immunization with live murine pneumonia virus-vectored SARS-CoV-2 vaccine is protective in macaques. *Res Sq*, doi:10.21203/rs.3.rs-3278289/v1 (2023).

10 Le Nouën, C. *et al.* Intranasal pediatric parainfluenza virus-vectored SARS-CoV-2 vaccine is protective in monkeys. *Cell* **185**, 4811-+, doi:10.1016/j.cell.2022.11.006 (2022).

11 Ponce-de-Leon, S. *et al.* Interim safety and immunogenicity results from an NDV-based COVID-19 vaccine phase I trial in Mexico. *NPJ Vaccines* **8**, 67, doi:10.1038/s41541-023-

00662-6 (2023).
12 Grifoni, A. *et al.* SARS-CoV-2 human T cell epitopes: Adaptive immune response against COVID-19. *Cell Host Microbe* **29**, 1076-1092, doi:10.1016/j.chom.2021.05.010 (2021).
13 Moss, P. The T cell immune response against SARS-CoV-2. *Nat Immunol* **23**, 186-193, doi:10.1038/s41590-021-01122-w (2022).
14 Chandrashekar, A. *et al.* Vaccine protection against the SARS-CoV-2 Omicron variant in macaques. *Cell* **185**, 1549-1555 e1511, doi:10.1016/j.cell.2022.03.024 (2022).
15 Gray, G. *et al.* Effectiveness of Ad26.COV2.S and BNT162b2 Vaccines against Omicron Variant in South Africa. *N Engl J Med* **386**, 2243-2245, doi:10.1056/NEJMc2202061 (2022).
16 Gao, Y. *et al.* Ancestral SARS-CoV-2-specific T cells cross-recognize the Omicron variant. *Nat Med* **28**, 472-476, doi:10.1038/s41591-022-01700-x (2022).
17 Horner, C. *et al.* A highly immunogenic and effective measles virus-based Th1-biased COVID-19 vaccine. *Proc Natl Acad Sci U S A* **117**, 32657-32666, doi:10.1073/pnas.2014468117 (2020).
18 Frantz, P. N. *et al.* A live measles-vectored COVID-19 vaccine induces strong immunity and protection from SARS-CoV-2 challenge in mice and hamsters. *Nat Commun* **12**, 6277, doi:10.1038/s41467-021-26506-2 (2021).
19 Frantz, P. N., Teeravechyan, S. & Tangy, F. Measles-derived vaccines to prevent emerging viral diseases. *Microbes Infect* **20**, 493-500, doi:10.1016/j.micinf.2018.01.005 (2018).
20 Lu, M. *et al.* A safe and highly efficacious measles virus-based vaccine expressing SARS-CoV-2 stabilized prefusion spike. *Proc Natl Acad Sci U S A* **118**, doi:10.1073/pnas.2026153118 (2021).
21 Zhang, Y. *et al.* A highly efficacious live attenuated mumps virus-based SARS-CoV-2 vaccine candidate expressing a six-proline stabilized prefusion spike. *Proc Natl Acad Sci U S A* **119**, e2201616119, doi:10.1073/pnas.2201616119 (2022).
22 Mura, M. *et al.* hCD46 receptor is not required for measles vaccine Schwarz strain replication: Type-I IFN is the species barrier in mice. *Virology* **524**, 151-159, doi:10.1016/j.virol.2018.08.014 (2018).
23 Pickar, A. *et al.* Establishing a small animal model for evaluating protective immunity against mumps virus. *PLoS One* **12**, e0174444, doi:10.1371/journal.pone.0174444 (2017).
24 Cheng, L. Q., Wang, Y. & Du, J. Human Papillomavirus Vaccines: An Updated Review. *Vaccines-Basel* **8**, doi:ARTN 39110.3390/vaccines8030391 (2020).
25 Harper, D. M. & DeMars, L. R. HPV vaccines - A review of the first decade. *Gynecol Oncol* **146**, 196-204, doi:10.1016/j.ygyno.2017.04.004 (2017).
26 Zhai, L. & Tumban, E. Gardasil-9: A global survey of projected efficacy. *Antiviral Res* **130**, 101-109, doi:10.1016/j.antiviral.2016.03.016 (2016).
