# Supplementary material for: Three SARS-CoV-2 spike protein variants delivered intranasally by measles and mumps vaccines are broadly protective
Source: Nat Commun. 2024 Jul 3;15:5589. doi: 10.1038/s41467-024-49443-2 (PMC11222507; doi:10.1038/s41467-024-49443-2)
Supplement: Supplementary file 3 — Description of Additional Supplementary Files [file 41467_2024_49443_MOESM3_ESM.docx]

**Description of Additional Supplementary Files**

**Supplementary Data 1: Codon optimized nucleotide sequence of preS-6P genes used in the study.** The spike genes are derived from SARS-CoV-2 USA-WA1/2020 (WA1) natural isolate (NR-52281, GenBank accession no. [MN985325](https://www.ncbi.nlm.nih.gov/nuccore/MN985325) ), SARS-CoV-2 Delta (B.1.617.2) (NR-55672, GISAID: EPI_ISL_2331496), SARS-CoV-2 Alpha (B.1.1.7) **(**NR-54000, GISAID: EPI_ISL_683466), Beta (B.1.351) (NR-55282, GISAID: EPI_ISL_890360)**,** and SARS-CoV-2 Omicron BA.1 (B.1.1.529) (NR-56461, GISAID: EPI_ISL_7160424). All spikes were codon optimized for expression in mammalian cells, the furin cleavage site was deleted to prevent the S1/S2 cleavage, the transmembrane/cytoplasmic tail (TM/CT) domain was replaced with a T4 fibritin self-trimerizing domain, and six proline substitutions (K986P, V987P, F817P, A892P, A899P, and A942P) were introduced to stabilize the protein.
