# Supplementary material for: Three SARS-CoV-2 spike protein variants delivered intranasally by measles and mumps vaccines are broadly protective
Source: Nat Commun. 2024 Jul 3;15:5589. doi: 10.1038/s41467-024-49443-2 (PMC11222507; doi:10.1038/s41467-024-49443-2)
Supplement: Supplementary file 5 — Reporting Summary [file 41467_2024_49443_MOESM5_ESM.pdf]

nature portfolio

Corresponding author(s): Jianrong Li

Last updated by author(s): 05/20/2024

Reporting Summary

Nature Portfolio wishes to improve the reproducibility of the work that we publish. This form provides structure for consistency and transparency in reporting. For further information on Nature Portfolio policies, see our Editorial Policies and the Editorial Policy Checklist.

Statistics

For all statistical analyses, confirm that the following items are present in the figure legend, table legend, main text, or Methods section.

n/a	Confirmed	
☐	☒	The exact sample size (*n*) for each experimental group/condition, given as a discrete number and unit of measurement
☐	☒	A statement on whether measurements were taken from distinct samples or whether the same sample was measured repeatedly
☐	☒	The statistical test(s) used AND whether they are one- or two-sided<br>*Only common tests should be described solely by name; describe more complex techniques in the Methods section.*
☒	☐	A description of all covariates tested
☐	☒	A description of any assumptions or corrections, such as tests of normality and adjustment for multiple comparisons
☐	☒	A full description of the statistical parameters including central tendency (e.g. means) or other basic estimates (e.g. regression coefficient) AND variation (e.g. standard deviation) or associated estimates of uncertainty (e.g. confidence intervals)
☐	☒	For null hypothesis testing, the test statistic (e.g. *F*, *t*, *r*) with confidence intervals, effect sizes, degrees of freedom and *P* value noted<br>*Give P values as exact values whenever suitable.*
☒	☐	For Bayesian analysis, information on the choice of priors and Markov chain Monte Carlo settings
☒	☐	For hierarchical and complex designs, identification of the appropriate level for tests and full reporting of outcomes
☒	☐	Estimates of effect sizes (e.g. Cohen's *d*, Pearson's *r*), indicating how they were calculated

Our web collection on statistics for biologists contains articles on many of the points above.

Software and code

Policy information about availability of computer code

Data collection	All Western blot images were developed and photographed by FluorChem Western blot imaging systems (Bio-Rad). All ELISA-based data were recorded by ELISA plate reader. The flow cytometry data of spleen T cells were collected using a BD LSRFortessa X-20 flow cytometer following compensation with UltraComp eBeads (Invitrogen). The flow cytometry data of lung T cells were collected on a Cytek Aurora flow cytometer (Cytekbio). Data were analyzed using FlowJo v10 (Tree Star). All viral titer data were quantified by plaque assay. Figures were generated with GraphPad Prism version 6.01. All original data were kept in a notebooks or electronically in lab computers.
Data analysis	Nucleotide and amino acid sequence analysis was performed using DNAstar Software (Lasergene, Madison, Wisconsin). All biological experiments were done three (n= 3) biologically independent experiments to warrant the samples size and statistical analysis, as standard for biological experiments. GraphPad Prism version 6.01 was used to generate figures. Flow cytometry data were analyzed using FlowJo v10 (Tree Star). Results were reported as means ± standard deviation, as indicated in the figures and figure legend. Western blot data are representative of n = 3 biologically independent experiments. Statistical analysis was performed by one-way or two way multiple comparisons using GraphPad Prism version 6.01 or two-sided Student's t test. A P value of <0.05 was considered statistically significant.*P<0.05, **P<0.01, ***P<0.001, ****P<0.0001.

For manuscripts utilizing custom algorithms or software that are central to the research but not yet described in published literature, software must be made available to editors and reviewers. We strongly encourage code deposition in a community repository (e.g. GitHub). See the Nature Portfolio guidelines for submitting code & software for further information.

Data

Policy information about availability of data

All manuscripts must include a data availability statement. This statement should provide the following information, where applicable:

- Accession codes, unique identifiers, or web links for publicly available datasets
- A description of any restrictions on data availability
- For clinical datasets or third party data, please ensure that the statement adheres to our policy

The experimental data generated in this study are provided in the main text, Figures, or in the Supplementary Information/Source Data File.

Research involving human participants, their data, or biological material

Policy information about studies with human participants or human data. See also policy information about sex, gender (identity/presentation), and sexual orientation and race, ethnicity and racism.

Reporting on sex and gender	No human participants, data, or biological material were used in this study.
Reporting on race, ethnicity, or other socially relevant groupings	No human participants, data, or biological material were used in this study.
Population characteristics	No human participants, data, or biological material were used in this study.
Recruitment	No human participants, data, or biological material were used in this study.
Ethics oversight	No human participants, data, or biological material were used in this study.

Note that full information on the approval of the study protocol must also be provided in the manuscript.

Field-specific reporting

Please select the one below that is the best fit for your research. If you are not sure, read the appropriate sections before making your selection.

☒ Life sciences ☐ Behavioural & social sciences ☐ Ecological, evolutionary & environmental sciences

For a reference copy of the document with all sections, see nature.com/documents/nr-reporting-summary-flat.pdf

Life sciences study design

All studies must disclose on these points even when the disclosure is negative.

Sample size	All results were from three (n = 3) biologically independent experiments to warrant the samples size and statistical analysis, as standard for biological experiments. For animal studies, group of 5 to 15 animals were used in each study. The sample size in animal studies was calculated according to previous report (Charan and Kantharia, The Journal of Pharmacology & Pharmacotherapeutics, 2013). Results were reported as means ± standard deviation, as indicated in the figures. Western blots and cell cytopathic images are representatives of three biologically independent experiments. Statistical analysis was performed by one-way or two way multiple comparisons using GraphPad Prism version 6.01 or two-sided Student's t test. A P value of <0.05 was considered statistically significant.*P<0.05, **P<0.01, ***P<0.001, ****P<0.0001.
Data exclusions	No data was excluded in the analysis.
Replication	All experiments include cloning, DNA ligation, Western blot, viral growth kinetics, ELISA, serum IgG, IgA, neutralization antibody detection, flow cytometry and in vivo experiments have been successfully replicated three times, yielding consistent results. The results were reliably reproduced.
Randomization	All samples/organisms were randomly assigned to experimental groups. Data were also collected randomly. Bulk stocks of plasmids, antibody, virus stocks, and other reagents were prepared for each experiments. The cells were randomly assigned to each treatment group. Animal experiments were randomized (see below for detail).
Blinding	Investigators were blinded to group allocation for animal experiments, including virus inoculation, tissue collection, processing, virology analysis, and evaluation of histologic lesions. Investigators were also blinded to for in vitro experiments during data collection and analysis.

Reporting for specific materials, systems and methods

We require information from authors about some types of materials, experimental systems and methods used in many studies. Here, indicate whether each material, system or method listed is relevant to your study. If you are not sure if a list item applies to your research, read the appropriate section before selecting a response.

Materials & experimental systems

n/a	Involved in the study
	☒ Antibodies
	☒ Eukaryotic cell lines
☒	Palaeontology and archaeology
	☒ Animals and other organisms
☒	Clinical data
☒	Dual use research of concern
☒	Plants

Methods

n/a	Involved in the study
☒	ChIP-seq
	☒ Flow cytometry
☒	MRI-based neuroimaging

Antibodies

Antibodies used

The below antibodies were used for Western blot.

Rabbit anti-SARS-CoV-2 N-protein monoclonal antibody: SinoBiological, Cat. #40143-R001, 1:5,000
Rabbit anti-SARS Nucleocapsid protein: Novus Biologicals, Cat. #NB100-56576, 1:5,000
HRP-labeled goat anti-rabbit secondary antibody: Invitrogen, Cat. #A27036, 1:5,000
Rabbit anti-SARS-CoV-2 S-protein monoclonal antibody: SinoBiological, Cat. # Cat: 40150-R007, 1:2,000
Mouse anti-SARS-CoV-2 S-protein monoclonal antibody: SinoBiological, Cat. # 40591-MM42; 1:2000
Beta-actin: Proteintech, 66009, dilution: 1:5000

The following antibodies for spleen T cell assay were purchased from Biolegend (clone, catalog number, dilution):
CD3-PE/Cyanine7 (145-2C11, 100319, 1:400),
IFNγ-PE/Dazzle 594 (XMG1.2, 505845, 1:400),
TNFα-Brilliant Violet 785 (MP6-XT22, 506341, 1:400),
CD107a-Alexa Fluor 488 (1D4B, 121607, 1:400),
granzyme-B-Alexa Fluor 647 (GB11, 515405 1:200),
IL-4-Brilliant Violet 711 (11B11, 504133, 1:100),
CD4-BUV 496 (GK1.5, 612952, 1:400),
CD8-BUV737 (53-6.7, 612759, 1:400),
IL-10-Brilliant Violet 510 (JES5-16E3, 563277, 1:100),
IL-2-PE (JES6-5H4, 12-7021-82, 1:200).

Below antibodies used for lung T cell assay:
CD3 V450 (clone 17A2, 1:500 dilution, BD Biosciences, catalog no. 561389),
CD4 BV750 (clone H129.19, 1:1,000 dilution, BD Biosciences, catalog no. 747275),
CD44 PerCP-Cy5.5 (clone IM7, 1:500 dilution, BD Biosciences, catalog no. 560570),
CD62L BV605 (clone MEL-14, 1:2,000 dilution, BD Biosciences, catalog no. 563252),
CD69 BV711 (clone HI.2F3, 1:500 dilution, BD Biosciences, catalog no. 740664),
IFN-g FITC (clone XMG1.2, 1:125 dilution, eBioscience, catalog no. 11-7311-82),
IL-17 PE-Cy7 (clone eBio17B7, 1:125 dilution, eBioscience, catalog no. 25-7177-82),
IL-5 APC (clone TRFK5, 1:125 dilution, BD Biosciences, catalog no. 554396),
CD8 APC (clone 53-6.7, 1:1,000 dilution, BioLegend, catalog no. 100712).

Validation

For antibodies used for protein detection, we carried out Western blot according to the method on the company's website and instruction, and determined whether the size of the protein band met the expected molecular weight. Appropriate positive control and negative control, and protein markers were used for validation. The antibodies used for T cell assay were purchased from commercial companies (Biolegend, BD Biosciences, or eBioscience) and have been validated by these companies. The specificity of these antibodies with appropriate positive and negative controls was also validated using intracellular antibody staining followed by flow cytometry analysis.

Eukaryotic cell lines

Policy information about cell lines and Sex and Gender in Research

Cell line source(s)

Vero CCL81 cells (African green monkey, ATCC no. CCL81), Vero E6 cells (ATCC CRL-1586), and HEp-2 cells (ATCC no. CCL-23) were purchased from American Type Culture Collection (ATCC, Manassas, VA). FreeStyle293F cells (Catalog no. R79007) were purchased from Thermo Fisher (Waltham, MA, USA).

Authentication	Vero CCL81, Vero E6, and HEp-2 cells were purchased from ATCC. According to ATCC's instruction, the cell lines have been authenticated using STR profiling before shipped to us. FreeStyle293F cells were authenticated using STR profiling by Thermo Fisher. We first made a bulk stocks for each cell line after recovering from the original frozen vial from ATCC. We will discard the cells after passing 15-20 passages, and recover new cells from frozen stocks. Cell morphology was monitored at each passage by microscope. Periodically, growth curve analysis for cell line was performed.
Mycoplasma contamination	Periodically, all cell lines in our lab were tested for contamination of mycoplasma by the LookOut Mycoplasma PCR Detection Kit (Sigma, MP0035-1KT). All cell lines used in this study were free of mycoplasma.
Commonly misidentified lines (See ICLAC register)	No commonly used misidentified cell lines were used.

Animals and other research organisms

Policy information about studies involving animals; ARRIVE guidelines recommended for reporting animal research, and Sex and Gender in Research

Laboratory animals	All animals were housed at The Ohio State University ULAR animal facility at temperature of 22 degree C with 53% humidity. Light dark cycle is 12 hours light and 12 hours dark. Specific-pathogen-free (SPF) IFNAR1-/- mice were purchased from Jackson Laboratories (Bar Harbor, ME). Both female and male IFNAR1-/- mice were used for the studies. 4-6-week-old female golden Syrian hamsters were purchased from Envigo (Indianapolis, IN). All animals were housed in BSL-2 or BSL3 animal care facility at OSU, which is AAALAC accredited. For animal experiments, groups of 5 to 15 animals were randomly formed. Each group was inoculated with respective virus strain or PBS. All analysis (such as tissue collection, processing, virus detection, histologic evaluation) were blinded to researchers. Antibody titers and viral titers are the geometric mean titer (GMT) of 5 to 15 animals ± standard deviation, and were statistically analyzed.
Wild animals	No wild animals were used in the study.
Reporting on sex	For IFNAR1-/- mice, both female and male were used in the study. In Animal Experiments 1 and 3: each group (n=5) contains 3 female and 2 male mice. In Animal Experiment 2: Groups 1, 2, and 4 contain 10 mice (n=10), 5 females and 5 males; Group 3 contains 9 mice (n=9), 5 females and 4 males). Female hamsters were used in this study (Animal Experiments 4-8).
Field-collected samples	No field collected samples were used in the study.
Ethics oversight	All animals were housed within ULAR facilities of The Ohio State University under approved Institutional Animal Care and Use Committee (IACUC) guidelines (protocol no. 2009A0183 and 2020A00000053). Each inoculation group was separately housed in rodent cages under animal biosafety level 2 (BSL-2 for rMeV and rMuV) or BSL3 (for SARS-CoV-2) conditions. All experiments with infectious SARS-CoV-2 were conducted under biosafety level 3 (BSL3) at The Ohio State University and were approved by the Institutional Biosafety Committee (IBC).

Note that full information on the approval of the study protocol must also be provided in the manuscript.

Plants

Seed stocks	No plants were used in the study
Novel plant genotypes	No plants were used in the study
Authentication	No plants were used in the study

Flow Cytometry

Plots

Confirm that:

- [ ] The axis labels state the marker and fluorochrome used (e.g. CD4-FITC).
- [ ] The axis scales are clearly visible. Include numbers along axes only for bottom left plot of group (a 'group' is an analysis of identical markers).
- [ ] All plots are contour plots with outliers or pseudocolor plots.
- [x] A numerical value for number of cells or percentage (with statistics) is provided.

Methodology

Sample preparation	For T cell assay: Lung or spleens of immunized IFNAR-/- transgenic mice were aseptically removed after immunization and minced by pressing through cell strainers. Red blood cells were removed by incubation in 0.84 % ammonium chloride and, following a series of washes in RPMI 1640, cells were resuspended in RPMI 1640 supplemented with 2 mM l-glutamine, 1 mM sodium pyruvate, 10 mM HEPES, 100 U/ml penicillin, 100 µg/ml streptomycin, and 10% fetal calf serum. Spleen or lung T cells were restimulated in vitro with the antigen for 2-5 days. Cells were subjected to extracellular staining with lineage-specific antibodies and, after fixation, to intracellular staining with cytokine-specific antibodies.
Instrument	BD LSRFortessa X-20 flow cytometer (BD) for spleen T cell assay, Cytek Aurora flow cytometer (Cytekbio) for lung T cell assay
Software	FlowJo v10 was used to collect and analyze the data.
Cell population abundance	For detection of SARS-CoV-2-specific intracellular cytokine production, 10^6 cells were stimulated in 96-well round bottom plates with peptide pool (5 µg/ml), media alone or PMA/Ionomycin (BioLegend) as negative and positive controls, respectively, for 5-h in the presence of GolgiPlug (BD Biosciences). Following incubation, cells were surface stained for CD3, CD4, and CD8 for 30 min at 4°C, fixed and permeabilized using the cytofix/cytoperm kit (BD Biosciences), and intracellularly stained for IFNγ, TNFα, IL-2, Granzyme B, IL-10 & IL-4 for 30 min at room temperature. Dead cells were removed using the LIVE/DEAD fixable Near-IR dead cell stain kit (Invitrogen). A positive response was defined as >3 times the background of the negative control sample. The percentage of cytokine positive cells was then calculated by subtracting the frequency of positive events in negative control samples from that of test samples.
Gating strategy	Gating strategy for flow cytometry analysis. Spleen cells were restimulated in vitro with the antigen for 2-5 days. Cells were subjected to extracellular staining with lineage-specific antibodies and, after fixation, to intracellular staining with cytokine-specific antibodies. For flow cytometry analysis, cells were first gated on singlets and then live cells were selected (R1) based the forward (FSC) and side scatters (SSC). Live cells were then analyzed for expression of CD4 versus CD3 and the double positive population was identified as CD4+ T cells (CD3+CD4+). The frequency of cells producing a given cytokine among CD3 +CD4+ cells was determined by analyzing the number cells cytokine positive cells (R2). Events were collected on a BD LSRFortessa X-20 flow cytometer following compensation with UltraComp eBeads (Invitrogen). For lung T cell assay, samples were collected on a Cytek Aurora flow cytometer (Cytekbio). Data were analyzed using FlowJo v10 (Tree Star). Examples of gating strategy is shown in Fig.S17, S18, S19, S20, and S21.

☒ Tick this box to confirm that a figure exemplifying the gating strategy is provided in the Supplementary Information.
